# Promising Drug Delivery Approaches to Treat Microbial Infections in the Vagina: A Recent Update

**DOI:** 10.3390/polym13010026

**Published:** 2020-12-23

**Authors:** Manisha Pandey, Hira Choudhury, Azila Abdul-Aziz, Subrat Kumar Bhattamisra, Bapi Gorain, Teng Carine, Tan Wee Toong, Ngiam Jing Yi, Lim Win Yi

**Affiliations:** 1 Department of Pharmaceutical Technology, School of Pharmacy, International Medical University, Bukit Jalil, Kuala Lumpur 57000, Malaysia; 2 Centre for Bioactive Molecules and Drug Delivery, Institute for Research, Development and Innovation, International Medical University, Kuala Lumpur 57000, Malaysia; 3 Department of Chemical and Environmental Engineering, Malaysia-Japan International Institute of Technology, Universiti Teknologi Malaysia, Jalan Sultan Yahya Petra, Kuala Lumpur 54100, Malaysia; azila@ibd.utm.my or; 4 Department of Life Sciences, School of Pharmacy, International Medical University, Bukit Jalil, Kuala Lumpur 57000, Malaysia; subratkumar@imu.edu.my; 5 Faculty of Health and Medical Sciences, School of Pharmacy, Taylor’s University, Subang Jaya, Selangor 47500, Malaysia; bapi.gn@gmail.com; 6 Center for Drug Delivery and Molecular Pharmacology, Faculty of Health and Medical Sciences, Taylor’s University, Subang Jaya, Selangor 47500, Malaysia; 7 Undergraduate School of Pharmacy, International Medical University, Bukit Jalil, Kuala Lumpur 57000, Malaysia; TENG.CARINE@student.imu.edu.my (T.C.); TAN.WEETOONG@student.imu.edu.my (T.W.T.); NGIAM.JINGYI@student.imu.edu.my (N.J.Y.); LIM.WINYI@student.imu.edu.my (L.W.Y.)

**Keywords:** vaginal delivery, nanotechnology, mucoadhesive, stimuli-responsive, antimicrobial, infection

## Abstract

An optimal host–microbiota interaction in the human vagina governs the reproductive health status of a woman. The marked depletion in the beneficial *Lactobacillus* sp. increases the risk of infection with sexually transmitted pathogens, resulting in gynaecological issues. Vaginal infections that are becoming increasingly prevalent, especially among women of reproductive age, require an effective concentration of antimicrobial drugs at the infectious sites for complete disease eradication. Thus, topical treatment is recommended as it allows direct therapeutic action, reduced drug doses and side effects, and self-insertion. However, the alterations in the physiological conditions of the vagina affect the effectiveness of vaginal drug delivery considerably. Conventional vaginal dosage forms are often linked to low retention time in the vagina and discomfort which significantly reduces patient compliance. The lack of optimal prevention and treatment approaches have contributed to the unacceptably high rate of recurrence for vaginal diseases. To combat these limitations, several novel approaches including nano-systems, mucoadhesive polymeric systems, and stimuli-responsive systems have been developed in recent years. This review discusses and summarises the recent research progress of these novel approaches for vaginal drug delivery against various vaginal diseases. An overview of the concept and challenges of vaginal infections, anatomy and physiology of the vagina, and barriers to vaginal drug delivery are also addressed.

## 1. Introduction

Vaginal infection is a global health issue commonly seen in women at reproductive age. It generally includes bacterial vaginosis (BV), vulvovaginal candidiasis (VVC), and trichomoniasis [1,2]. It is defined as vaginal microbiota dysbiosis where one or more microbes are dominant to the other microbes present in the vagina [3]. The symptoms of vaginal infection include abnormal vaginal discharge, burning sensation, itching, irritation, and discomfort. Nevertheless, some women with vaginal infection present with fewer symptoms whereas some cases were found to be asymptomatic [1,2]. In 2019, BV was the most prevalent vaginal infection and it was estimated to affect 5% to 70% of the women worldwide [4]. Studies in 2018 stated that there were around 30% of women aged 14 to 49 were affected in the US [5,6]. The data also mentioned that women who began to engage in sexual intercourse activity at a young age, are commercial sex workers, are unmarried, or had multiple sex partners are more prone to BV [4]. In a healthy vagina, *Lactobacillus* sp. is present predominantly and it is responsible for producing lactic acid through hydrogen peroxide production in order to maintain the acidity of the vagina. This acidic environment protects the vagina by suppressing the growth of other microorganisms. BV has occurred when the *Lactobacillus* sp. is replaced by other vaginal flora such as *Staphylococcus* sp., *Peptostreptococcus* sp., *Gardnerella vaginalis*, *Mycoplasma hominis*, and the Enterobacteriaceae [7]. The prevalence of vaginal infections varies based on different demographic parameters. A study in Ethiopia showed a contrary result where candidiasis instead of BV was the most common vaginal infection followed by trichomoniasis. Candidiasis is mostly caused by *Candida* sp. whereas *Trichomonas vaginalis* infection causes trichomoniasis. This study also found that the prevalence of vaginal infections was comparatively higher in non-pregnant women than pregnant women. Moreover, they found that women at the age of 40 and above were more susceptible to BV due to the reduction of oestrogen thus altering the living environment of the *Lactobacillus* sp. [1]. Although vaginal infection may be asymptomatic or present with mild symptoms, the untreated vaginal infection can result in severe gynaecologic and obstetric complications. It also greatly increases the risk of acquiring sexually transmitted infections (STIs) such as acquired immunodeficiency syndrome (AIDS) [7].

Given the high vaginal infections prevalence globally, systemic and local treatments for vaginal problems have been practiced for a long time. Current therapeutic approaches to the management of vaginal infections include oral and topical broad-spectrum antibiotics or other antimicrobial agents specifically targeting the known causative microorganisms. In recent years, topical approaches have been deemed to be more favourable as it can achieve a higher local drug concentration by avoiding first-pass metabolism and has lesser side effects. Intravaginal dosage forms that are available in the market are tablets, capsules, pessaries, creams, ointments, and gels. However, conventional vaginal products are associated with a few limitations such as poor adhesive property and short retention time due to the self-cleansing mechanism of the vagina [8]. Therefore, various novel approaches had been studied by researchers to improve the drug-delivery system.

**Figure 1:**
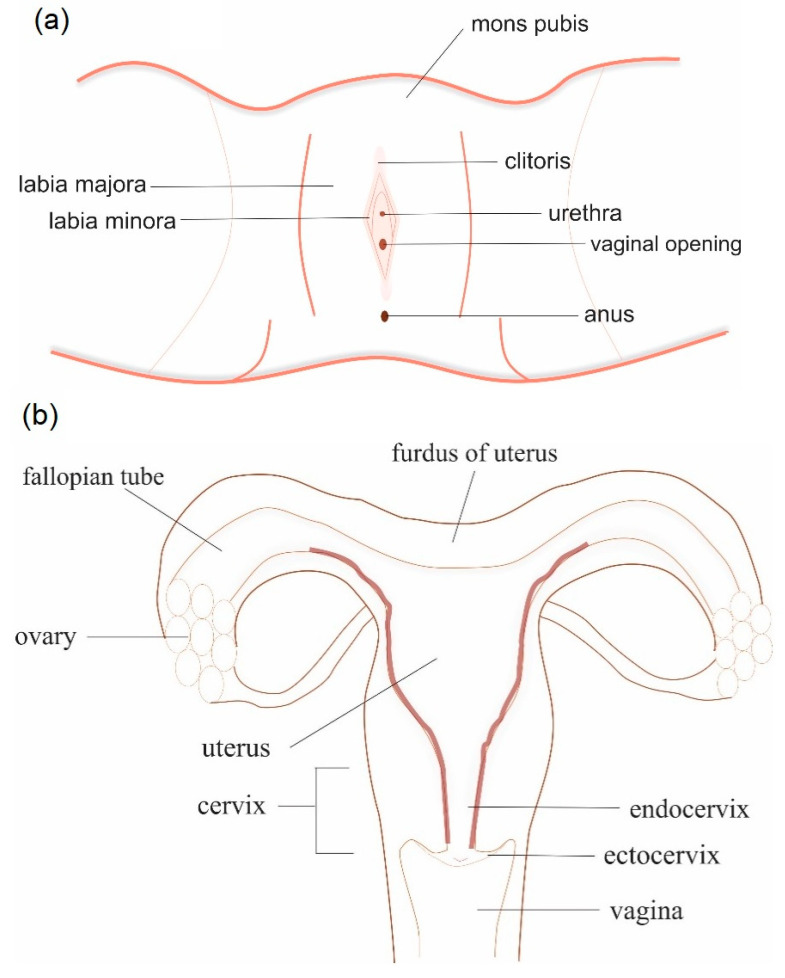
The illustration of the female external genitalia (**a**) and reproductive tract (**b**).

The female reproductive tract is categorized into the upper and lower part. The vagina is a part of the female lower reproductive tract together with the ectocervix and the external genitalia (mons pubis, labia majora, labia minora, clitoris and the vestibule of the vagina) (Figure 1a) [9,10]. The ectocervix is the end portion of the uterus that attaches to the vagina while the endocervix is the mucosa of the cervix (Figure 1b) [9]. The vagina is structured as a collapsed fibromuscular tube lined by stratified squamous epithelium [10]. Lactobacilli are found in the healthy vagina to provide further protection to the vagina mucosa against harmful microorganisms by producing lactic acid, hydrogen peroxide and bacteriocin-liked substances [10]. The mucus consistency and the thickness of the vaginal epithelium vary throughout the menstrual cycle and are regulated by the sex hormones such as oestrogen. It consists of 28 cell layers on the first 12 days of the cycle then reduces to 26 cell layers with a thickness of 261 ± 16 μm in the following week. Generally, the pH value of the vagina is between 3.5 and 5.5. In pregnant women, the pH of the vagina alters to around 3.8 to 4.4 during pregnancy and increases to 7.0 to 7.4 after menopause [11].

The vagina is an imperative alternative site to deliver drugs locally and systemically, attributed to the high exposure of the contact surface, dense vascularization, and avoidance of gastrointestinal environment and hepatic first-pass effect [12,13,14]. However, some concerns have arisen regarding the eligibility of the vagina as a route for administration due to its highly variable microenvironment contributed to by the alterations in vaginal microbiota and the chemical changes such as pH, vaginal fluids, and vaginal mucus [12,14,15]. This poses a challenge for the development of vaginal products.

**Figure 2:**
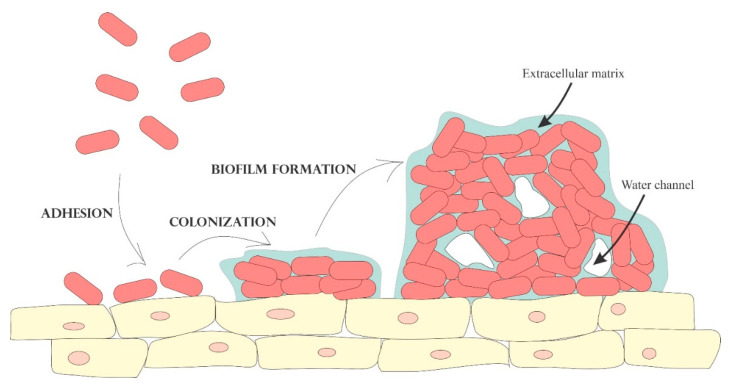
Schematic illustration of biofilm formation.

The vaginal microbiota forms a mutualistic relationship with the host and has vast significance in maintaining vaginal health and keeping the host against diseases. *Lactobacillus* sp. is determined to be the predominant microorganism in vaginal flora which renders the vaginal environment inhospitable to other pathogens by competing for nutrients and producing lactic acid [12,14,16]. Vaginal infections are the result of an imbalance in normal vaginal microbiota. Vaginal dysbiosis promotes the colonization of pathogens in the vagina and subjects to bacterial biofilm formation. Biofilm involvement implies the chronicity and recurrence of the disease [12,16]. Biofilm formed secondary to adaptation under diverse nutritional and environmental conditions is composed of a group of pathogenic microbes adhered to one another on a surface enclosed by the self-produced extracellular polymeric substances, as illustrated in Figure 2 [16]. The extracellular matrix acts as a physical barrier that limits the accessibility of antimicrobial molecules and evades the immune response [12]. Consequently, drug release will be altered and the pathogenic microbes have an increased persistence in a hostile environment and enhanced tolerance to the adverse conditions [12,16,17]. Moreover, some antimicrobials are unable to exert their action due to the reduced metabolism within the biofilm [12]. Hence, formulations indicated for vaginal delivery must not harm the normal vaginal flora and its environment.

Despite the additional advantages possessed by the vaginal route of administration, the pH, volume and viscosity of the vaginal fluid can influence drug absorption. Drug absorption is required to ensure therapeutic action is exerted at the targeted site. The extent of drug absorption is regulated by the ionization state of drugs, governed by the pH at the application site [12,14]. However, an altered vaginal pH is reported with vaginal microbial infections that affect the ionization of drug molecules, thereby modifying the drug absorption profile.

The cervicovaginal mucus (CVM) mixture is also known as the vaginal fluid is constituted by cervical mucus and cervicovaginal fluid [13]. Vaginal fluid acts as a barrier to protects the vagina against infection by trapping the pathogenic microbes [12,13]. However, the properties of the vaginal fluid vary by age, vaginal flora, vaginal practices, sexual intercourse, pathological conditions and throughout the menstrual cycle [12,14,15]. Variation in drug response arises due to the solubility of the drug affected by the water content in the vaginal fluid [12]. Eventually, poorly water-soluble drugs will solubilize in vaginal fluid when there is higher water content, and more will be absorbed.

The self-cleaning mechanism of the vagina cleans and keeps the vagina healthy by utilizing its natural secretions, which is the vaginal fluid, to maintain its pH [12]. However, drugs applied via the vagina route are eliminated by this mechanism. This limits the vaginal retention and influences therapeutic efficacy. For instance, conventional vaginal preparations are known to reside in the vagina cavity for a short duration owing to this physiological removal mechanism. Therefore, various polymers should be utilized to develop formulations with enhanced drug permanence onto vagina mucosa.

Polymer selection play a significant role in the release mechanism of drug from formulation, which will directly impact the therapeutic efficacy of formulation. An ideal characteristic of polymers for vaginal delivery is that they are biocompatible, do not absorb into mucus, should not irritate the mucous membrane, adhere quickly with mucin for sustainable release, and are stable in vaginal fluid. Both natural and synthetic polymers are extensively used to fabricate a vaginal drug-delivery system. Both types of polymer have their own benefits; however, a natural polymer has distinct benefits over a synthetic polymer such as being biocompatible, cost effective and easily modified for ligand conjugation. On the other hand, a synthetic polymer has no variation in their properties and better scale-up from research and development to production. Poly (lactic-co-glycolic acid) (PLGA) polyethers, polyacrylates, polyesters, poloxamers etc. are widely used polymers in fabricating carriers for vaginal drug delivery [18,19,20].

Considering all these factors in formulating an ideal vaginal drug-delivery tool is a challenge, which could alter the drug release profiles at the site of application and thereby the efficacy of the delivered drug. Hence, judicious selection of the different factors must be performed in the development of vaginal formulations. Therefore, the objectives of this review are to demonstrate advantages and challenges associated with the formulations, to critically appraise different optimization parameters based on their physicochemical properties, the in vitro release pattern, the in vivo efficacy and safety profile, and also to critically appraise the regulatory perspective of the formulations of different novel approaches for local delivery of antimicrobials for vaginal infection.

## 2. Type and Treatment of Vaginal Infections

The next sections describe the general concepts related to different types of vaginal infection, currently approved treatments, and the key challenges in the development of effective treatments.

Vaginal infections have been recognised as a global reproductive health issue that has affected a high number of women at reproductive age. Most of the reported cases are of microbial origin such as pathogenic bacteria, parasites, fungi, or viruses. These vaginal infections are associated with several discomfort and complications that reduce the self-esteem and quality of life of the women. The common types of infection include BV, VVC, trichomoniasis, human immunodeficiency virus (HIV) infection and human papillomavirus (HPV) infection [11].

BV is characterised by an overgrowth of anaerobic and microaerophilic bacteria, including *Gardnerella vaginalis*, *Atopobium vaginae*, *Bacteroides* spp., etc. [21,22,23]. Being the most prevalent form of vaginitis, BV shows a high global prevalence, ranging from 23% to 29% across regions [24]. Risk factors for developing BV include douching, sexual intercourse, and poor personal hygiene. Furthermore, BV can be symptomatic or asymptomatic. Infected women usually show symptoms such as thin white vaginal discharge with fishy odour, itchiness, and irritation [21,22,25]. Patients are exposed to complications such as adverse obstetric outcomes and risk of acquiring STIs [22,23,26]. Generally, BV is diagnosed using the Nugent criteria, Amsel criteria, or Hay–Ison criteria [22,26]. The recommended antimicrobial formulations are oral metronidazole, oral clindamycin, oral tinidazole, metronidazole gel, clindamycin cream and clindamycin ovules [27,28,29,30].

VVC is a fungal infection caused primarily by *Candida albicans*. It is estimated that nearly 75% of women will experience at least one episode of VVC in their lifetime [31,32,33]. The high occurrence has been related to a patient’s sexual and hygienic habits, the use of hormones and antibiotics, pregnancy, and immunosuppression. Symptoms of VVC include abnormal vaginal discharge, dysuria, dyspareunia, and vaginal soreness [31,34]. Moreover, the management of VVC is mainly indicated for women with symptomatic infection. Most of the patients respond favourably to oral and topical azole therapies such as fluconazole, clotrimazole, miconazole, tioconazole, butoconazole and terconazole [32,34,35,36].

Trichomoniasis is caused by a protozoan parasite, *Trichomonas vaginalis*. It is a widespread non-viral STI that affected around 5.3% of women worldwide in 2016, with the majority of the cases being asymptomatic [37,38]. In symptomatic cases, patients may present with yellow-green vaginal discharge, lower abdominal pain, dysuria, and vulvar irritation [11,37]. The infection has been associated with infertility, poor pregnancy outcomes and STIs acquisition [11,37,39]. The mainstay treatment for trichomoniasis is oral metronidazole and tinidazole. Topical formulations are not recommended as they are often insufficient for complete disease eradication, resulting in lower cure rates as compared to the oral formulations [40,41].

HIV infection is one of the commonest viral STIs. It is estimated that approximately 38 million people were living with HIV at the end of 2019 [42]. Populations who are at elevated risk of infection are intravenous drug users, sex workers, transgender people and men who have sex with men [42,43]. HIV-infected patients usually develop symptoms such as fever, myalgias, and swollen lymph nodes. Additionally, the infection contributes to complications such as liver dysfunction, tuberculosis, and AIDS [43,44,45,46]. In Malaysia, the available antiretroviral drugs are: (1) nucleoside or nucleotide reverse transcriptase inhibitors (e.g., tenofovir); (2) non-nucleoside reverse transcriptase inhibitors (e.g., nevirapine); (3) protease inhibitors (e.g., ritonavir); (4) integrase inhibitors (e.g., raltegravir); (5) CCR5 antagonist (e.g., maraviroc), and (6) fusion inhibitors (e.g., enfuvirtide) [47].

HPV infection among the vaginal conditions is another common form of viral invasion that resulted in nearly 84% of new cases worldwide in 2018 [48]. Invasion of HPV occurs in the cervical squamous epithelium, particularly the basal layer. The viral particles are retained in the form of episomes in the basal layer, where numbers of virions are increased through the differentiation of epithelial cells [44]. As the initial response to the acute infection of HPV is mediated by the antimicrobial peptide producing epithelial cells and mucosal natural killer cells, the majority of patients experience mild symptoms during the initial stage of HPV infection such as the development of genital warts. However, infection of high-risk HPV can escape the adaptive and innate immune system of the body. The long-lasting infection causes patients to be vulnerable to implications, including cervical cancer, vaginal cancer and oropharyngeal cancer [44,49]. According to the World Health Organisation (WHO), vaccination is the most effective approach in cervical cancer prevention. Cryotherapy is suggested in the management of precancerous lesions [50,51].

Despite the availability of treatment, persistent or recurrent infection remains the greatest challenge of vaginal infections [26,33,47,52,53]. This reflects a possibility of treatment failure or reinfection which may be due to the following factors: (1) the presence of antimicrobial-resistant strains; (2) sharing common clinical presentations with other form of vaginitis, leading to misdiagnosis, or (3) poor patient adherence resulting from the limitations of available formulations [22,52,54,55,56,57,58,59,60]. All information has been summarised in Table 1.

## 3. Novel Approaches for Vaginal Drug Delivery for Microbial Infections

The vagina has been the site for local delivery of various therapeutic agents including antimicrobial agents, spermicides, contraceptives, and labour inducers. Most vaginal dosage forms can be self-administered easily with minimal interference to daily life [61,62]. However, these dosage forms have been proven to be associated with multiple limitations including the inadequate spreading of drugs over vaginal surfaces and low drug penetration to the submucosal layers. The possibility of leakage and expulsion of semi-solid dosage forms, due to vaginal fluid dynamics and self-cleaning action of the vaginal tract, may lead to incomplete delivery of sufficient doses. As a result, multiple doses are often needed daily to achieve the desired therapeutic effects, resulting in poor patient compliance and increased overall treatment costs [61,62]. Additionally, the poor ability of these dosage forms to modulate the diffusion of drugs within the CVM poses permeability issues [63]. Moreover, vaginal drug delivery has demonstrated inter-individual variations in the extent and rate of drug absorption due to the differences in physiological characteristics such as vaginal pH, cervical mucus, microbial flora, and cyclic changes during menstruation [64].

To overcome these drawbacks, several novel approaches have been developed for efficient vaginal drug delivery. An ideal vaginal drug delivery system (VDDS) should distribute uniformly throughout the vaginal cavity, retain at the administration site for a prolonged time, and provide sustained drug release without causing local side effects to the vaginal epithelium [8,61]. The following sections will discuss the various studies done by researchers on the nanocarriers and mucoadhesive polymeric approaches in vaginal drug delivery for different types of vaginal infection.

### 3.1. Nanocarriers

Nanostructures offer compelling future prospects in drug delivery, in view of the appealing features such as extended and controlled release, mucus adhesion or modulation and precise targeting ability for mucosal and intracellular delivery [65]. In addition, studies reported less association of side effects with nanostructured systems containing lower drug doses without compromising their therapeutic activity [14,15,66,67,68,69]. Hence, various application of these nanocarriers have been explored for VDDS to overcome the limitations of the conventional formulations.

Liposomes are of particular interest in terms of targeting ability and safety issues due to their phospholipid composition and easy manipulation for different delivery considerations with their flexible physicochemical characteristics [70]. Vanić et al. developed azithromycin liposomes with different bilayer elasticity to explore their potential in treating cervicovaginal infections. Conventional liposomes (CLs) were reported to be superior in preventing the formation of biofilms with half maximal inhibitory concentration (IC_50_) values up to eight-fold lower than free azithromycin as the consequence of the bilayer properties of rigid liposomes that have a higher phase transition temperature allowing slow release of azithromycin. Deformable propylene glycol liposomes (DPGLs) showed the significant inhibitory effect on biofilms due to the use of propylene glycol and monoacyl phosphatidylcholine that enhanced liposomes delivery through the formed biofilms, generating high local concentration of azithromycin. Biocompatibility study performed showed a slightly increased sensitivity of HeLa cells towards DPGLs as a notable lower concentration of monoacyl phosphatidylcholine was used with a short exposure time (24 h), suggesting less cytotoxic. Hence, it can be concluded that CLs is a potential dosage form for treatment of superficial infection and prevention of complicated infections while DPGLs are a promising approach for biofilm-related infections [71].

The high recurrence rate of HPV infections was associated with the failure to eradicate the virus and poor patient acceptance with the current available treatment options. However, mucosal delivery of interferon alpha-2b (IFN α-2b) are often limited by the presence of mucus. To enhance topical delivery of IFN α-2b for HPV vaginal infections, Jøraholmen et al. developed mucus-penetrating poly-ethylene glycol (PEG)-coated liposomes. The binding efficiency of PEGylated liposomes at various pH conditions was significantly lower than both conventional liposomes and chitosan-coated liposomes. In addition, an increase in interferon release for a prolonged period and a prominent penetration of interferon from PEGylated liposomes was shown in ex vivo testing. These findings confirmed the absence of interaction between PEGylated liposomes and mucin, and possible surface adsorption assured drug delivery is of immediate proximity to the vaginal epithelium [72].

Alternatively, pregnant females have higher vulnerability to vaginal infection including human simplex virus (HSV) due to the reduced activities of T-helper cell type 1 to protect the growing foetus. Growth and development of the foetus will be affected if the infected mother is not treated in time. However, pathogen resistance was found with the current anti-microbial options and the conventional course of anti-microbial therapy is inapplicable during pregnancy. Regarding both issues, Jøraholmen and colleagues developed mucoadhesive liposomes loaded with resveratrol using chitosan for vaginal inflammation and infections to improve the bioavailability, solubility and stability of resveratrol. Mucin studies performed at both healthy vaginal conditions and infected vaginal conditions revealed enhanced mucin binding properties with liposomes coated with a lower concentration of chitosan irrespective of vaginal pH conditions attributed to least interaction with liposomal surface that allows more surface available chitosan. Prolonged release of resveratrol from both coated and non-coated liposomes achieved from in vitro release studies and its superior antioxidant and anti-inflammatory properties suggested a potential vaginal approach [73].

On the other hand, polymeric nanoparticles (NPs) are the most extensively studied nanosystem for vaginal drug delivery. To ensure the desired therapeutic response is achieved, efforts are made to lengthen the residence time in the vaginal and improve their penetration through the mucosa. Martínez-Pérez et al. developed a PLGA nanoparticles surface modified with chitosan to deliver clotrimazole for vaginal delivery. It was demonstrated that the mucoadhesive properties of nanoparticles was enhanced upon addition of chitosan to its surface. This was owed to the greater affinity of cationic chitosan to the negatively charged mucin that further promoted polyelectrolyte complexes formation, enhancing mucoadhesion. Chitosan-modified PLGA nanoparticles showed a biphasic release profile with enhanced drug release rate up to 18 days relative to non-modified nanoparticles, offering single dose administration to maintain the microbicidal effect. Microbiological testing demonstrated a four-fold increase in antifungal activity inhibition against *Candida* sp. of clotrimazole when encapsulated in chitosan-modified nanoparticles due to the suitable size of nanoparticles that promote phagocytosis for cellular internalization of clotrimazole. On the other hand, this increment in antifungal effect was reported to be part of the microbicide properties of chitosan by binding with the negatively charged fungi surface that altered the permeability of the fungal cell wall and inhibited the ergosterol synthesis with clotrimazole. Cell viability greater than 80% at tested concentrations, revealing the biocompatibility and biosafety of the chitosan modified PLGA nanoparticles to vaginal epithelium [74].

In a similar approach, Amaral et al. designed a chitosan-based nanoparticle, where similar reduction in colony forming unit (CFU) levels, hyphae and cellular infiltrate were observed in the murine VVC models after a seven days treatment with miconazole in the form of both marketed cream and chitosan nanoparticles. However, this result was achieved with a seven-fold lower concentration of miconazole nitrate in chitosan-based nanoparticles relative to the concentration in conventional cream. The potentiated miconazole nitrate activity was due to the antifungal characteristic and mucoadhesive capacity of chitosan proved, respectively, with downregulation of TNFα and interleukins 10 (IL-10) seen in vaginal tissue with both empty and drug-loaded chitosan-based nanoparticles and the extended retention within the vaginal mucosa that assists drug release in a sustained manner. Similar levels of biochemical parameters of kidneys and liver and fractional DNA content in mice bone marrow cells were shown in all treatment groups, stipulating the absence of nephrotoxicity, hepatoxicity and genotoxicity with chitosan-based nanoparticles containing miconazole nitrate. These data indicate the feasibility of this formulation to emerge as a promising alternative for treatment of VVC [75].

**Figure 3:**
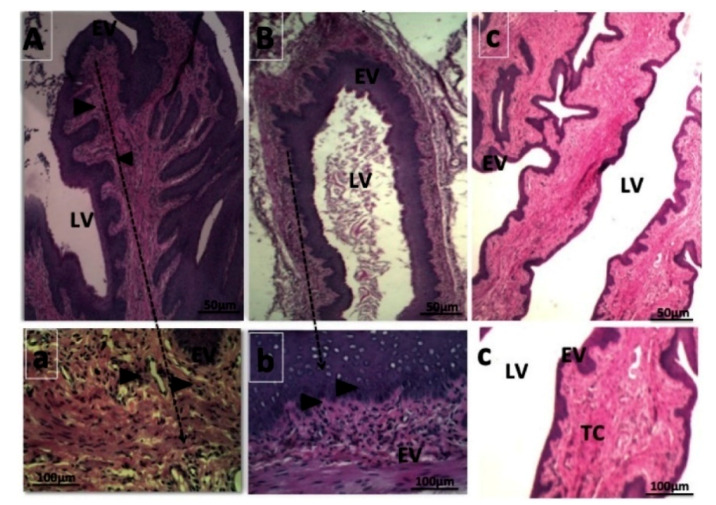
Histological sections of the endocervix collected 24 h post-infection. (**A**) Infected control receiving no treatment (Group 1). The vaginal lumen (LV) showed *Candida albicans* hyphae. The vaginal epithelium (EV) showed inflammatory cells. (**a**) Head arrow indicated the inflammatory infiltrate. (**B**) Infected and untreated animals receiving EUD (Eudragit) nanoparticles/hyaluronic acid (HA) (Group 2). The LV showed intense colonization by *Candida albicans*. The EV showed an inflammatory process. (**b**) Head arrow indicated the inflammatory infiltrate. (**C**) Infected and treated animals receiving AMP EUD nanoparticles/HA (Group 4). The LV showed no fungal infection and the inflammation was not evident. (**c**) The integrity of the EV and fibroelastic connective tissue (TC) after treating by AMP released from EUD nanoparticles/HA. Adapted from “Amphotericin B-loaded Eudragit RL100 nanoparticles coated with hyaluronic acid for the treatment of vulvovaginal candidiasis” by Melo [77].

A different polymer was used in a study where Melo et al. prepared eight formulations of AmB-loaded Eudragit RL100 nanoparticles (AMP EUD nanoparticles) to be optimized for treatment of VVC locally to overcome the associated systemic toxicity of AMP (e.g., nephrotoxicity and haemolytic anaemia). The optimized AMP-loaded nanoparticles were subsequently selected to be coated with hyaluronic acid (HA) and successfully characterized. Both coated and uncoated AMP EUD nanoparticles showed extended AMP release up to 81% for four days controlled by the limited swelling capacity of EUD RL100 and indicated HA as the coating polymer does not act as a barrier affecting drug release. In vitro antifungal activity evaluation illustrated a smaller inhibition zone induced by AMP released from HA-coated EUD nanoparticles compared to AMP in solution over 48 h due to the stepwise solubilization to displace the entrapped AMP. Complete eradication of fungal infection was reported in an animal treated with HA coated AMP EUD nanoparticles and uncoated AMP EUD nanoparticles after 24 h and further confirmed with the non-existence of *C. albicans* colonization and the absence of intense inflammation in the vaginal lumen illustrated in histological analysis (Figure 3). This accelerated elimination was due to the use of HA that was known as one of the major ligands of the CD44 receptor which was expressed on the vaginal epithelial cells, promoting the uptake of nanoparticles via receptor-mediated endocytosis and improving AMP bioavailability in the targeted area [76]. Moreover, adhesion of HA coated AMP EUD nanoparticles activated Toll-like receptors 2 and 4 that induced the release of various antimicrobial peptide and protective factors from the vaginal epithelium, facilitating the repair mechanism to preserve the epithelial barrier function which is important to prevent inflammation [77].

Calvo et al. studied the suitability of chitosan nanocapsules comprising of TIO and econazole (ECO) in vaginal application. A release assay performed in simulated vaginal fluid medium over two days reported 60% TIO release from TIO-loaded nanocapsules and 46% ECO release from ECO-loaded nanocapsules with respect to free TIO and ECO in solution. This demonstrated sustained release properties of nanocapsules relative to the free drug solutions. In vitro cytotoxicity on the human keratinocyte cell line (HaCaT) of drug-loaded nanocapsules were examined using an Methylthiazolyldiphenyl-tetrazolium bromide conversion (MTT) assay over a 24 h period. A relative cell viability of greater than 80% was reported with TIO-loaded and ECO-loaded chitosan nanocapsules at concentrations less than 12 µg/mL. Cell viability reduced with increasing concentrations of TIO and ECO up to 195 µg/mL and 95 µg/mL, respectively. This indicated similar effects with both drugs, and they were not cytotoxic at the concentrations tested. The MIC values against *C. albicans* of both chitosan nanocapsules were comparable with the drug solutions, suggested that the activity of both drugs was maintained throughout the nano-encapsulation process. Both drug-loaded nanocapsules presented fungicidal activity at non-toxic concentration (12 µg/mL) as both showed excellent time-to-kill profile with complete eradication of *C. albicans* culture in three days. Therefore, these results displayed the feasibility of this system for local treatment of vaginal candidiasis [78]. Finally, it is evident to mention that the exploration of delivering therapeutics against vaginal infection could effectively be monitored and controlled using this polymeric nanoparticular platform. However, modification in the fabrication process and delivery design are also improving day-by-day, where the formulated nanoparticles can be incorporated in some other tools (e.g., film or spray gel) for enhancing delivery options for patient compliance.

### 3.2. Vaginal Gels with Nanocarriers

**Figure 4:**
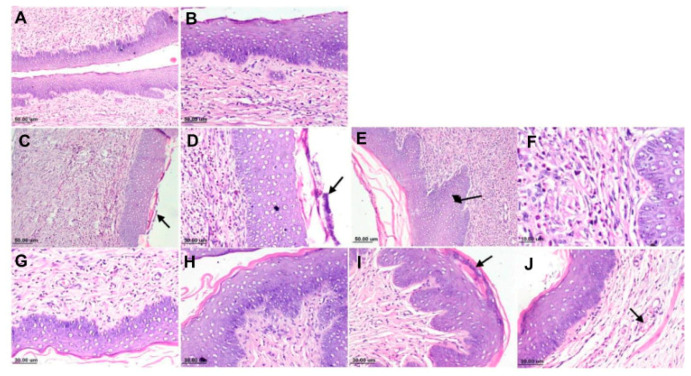
Photomicrograph of rat vagina, Haemotoxylin and Eosin (H&E) stain: (**A**) Negative control group, normal histology of rat vagina; (**B**) negative control group, higher magnification, showing stratified squamous epithelium with dense sub-epithelial connective tissue; (**C**) positive control group, showing heavy subepithelial inflammatory cells infiltration with necrotic debris over the mucosal surface (arrow); (**D**) positive control group, higher magnification, showing dissolution of keratin layer with presence of necrotic tissue debris; (**E**) positive control group, showing hyperplastic mucosa (arrow); (**F**) positive control group, showing sub-epithelial neutrophils and mononuclear infiltration; (**G**) mucoadhesive liposomal gel group, showing mild sub-epithelial inflammatory cells infiltration; (**H**) showing intact mucosa; (**I**) sertaconazole control gel, showing mucosa with presence of necrotic debris in the keratin layer and (**J**) showing mild sub-epithelial edema with dilated blood vessels (arrow), adapted from “Formulation and Characterization of Sertaconazole Nitrate Mucoadhesive Liposomes for Vaginal Candidiasis” by Abdellatif et al. [79].

Abdellatif et al. designed a mucoadhesive pectin coated-Sertaconazole-loaded liposomal gel as a strategy to prolong the retention of the formulation in the vaginal cavity for enhanced the drug absorption. A reduction in drug penetration through vaginal mucosa was observed compared to conventional gel due to the pectin coating that hindered its diffusion, which is desirable to minimize systemic exposure. Pectin coated liposomes displayed higher mucin adsorption with more retained in the vaginal tissues compared to the conventional gel attributed to the maximum shift in zeta potential of mucin to a less negative value upon neutralization with the positive charged mucoadhesive liposomes coated with 0.1% pectin. In vitro release assays showed that coating liposomes with increasing concentration of pectin are able to sustain sertaconazole release over eight hours, which is essential to improve patient adherence with less frequent application. A significant reduction in the mean number of CFUs and least histopathological change (Figure 4) evidenced with coated liposomes was assured by the anticandidal activity of dimethyldidodecylammonium bromide (DDAB), a cationic surfactant, its prolonged retention and sustained drug release [79].

In a more advanced way of delivering drugs, Tuğcu-Demiröz incorporated benzydamide hydrochloride (BNZ) loaded liposome into the mucoadhesive gel forming a lipogel for vaginal application. In this study, BNZ was loaded into the liposome, gel, and lipogel along with mucoadhesive polymers hydroxypropyl methylcellulose (HPMC) and Carbopol^®^ 974P. The highest mucoadhesion values were observed in the HPMC K100M gels. Liposome formulations showed the lowest values for work of mucoadhesion among all the formulations because no mucoadhesive polymers were present. The developed lipogel was more effective than liposomes as it exhibited approximately 5-fold stronger mucoadhesive strength. Hence, HPMC K100M was shown to be a good choice of vehicle to form a lipogel attributed to its mucoadhesive property that eventually prolonged the retention time of the formulation in the vagina [80]. Moreover, Kenechukwu et al. developed a mucoadhesive microgel encapsulating the miconazole nitrate (MN)-loaded solid lipid microparticles for local treatment of VVC and polycarbophil was employed as the mucoadhesive polymer [81]. As a result, the mucoadhesive strength of the developed formulation was high and the addition of MN slightly reduced the mucoadhesive strength. [81,82]. Controlled drug release property was observed in the presence of PEG 4000 and the effect increased proportionally with the concentration of PEG 4000. The authors stated that the developed formulation was non-irritant and more effective than Daktarin^®^ (a marketed MN cream) in suppressing the growth of *C. albicans*. This work showed remarkable benefits of the MN-loaded microgel, representing a promising therapeutic option for VVC [81]. Given that *T. vaginalis* can easily develop resistance to the existing antimicrobial therapy, a recent work of Osmari et al. had evaluated the local effect of indole-3-carbinol (I3C) encapsulated in Eudragit^®^ RS100 nanocapsules and free I3C on vaginal trichomoniasis. This study found that I3C was able to control the production of reactive oxygen species (ROS) and impair the cell signalling by its anti-inflammatory effect. Nanocapsulated-I3C (NC-I3C) had enhanced activity against the protozoa as it increased the protozoa uptake. Despite the advantages of NC-I3C, the NC-I3C suspension was inconvenient for vaginal application. Hence, gellan gum was added to form a semi-solid hydrogel of NC-I3C (HG-NC-I3C). HG-NC-I3C demonstrated improved mucoadhesion and was non-irritant to the chorioallantoic membrane. As a result, mucoadhesive HG-NC-I3C was considered as an alternative therapy for vaginal trichomoniasis [83]. As RVVC cases are often reported as a result of fluconazole resistance, Ismail et al. formulated a nanoemulsion-based mucoadhesive gel loaded with oxiconazole nitrate (OXZ), an imidazole antifungal agent against vaginal candidiasis, for vaginal application. The mucoadhesive polymers evaluated in this study were HPMC, sodium carboxymethylcellulose (NaCMC), xanthan gum, and Carbopol 934. All the formulated nanoemulsion gels had significantly higher mucoadhesive strength than the Tinox^®^ cream (a marketed OXZ cream) as the oil globules in the nanoemulsion gel can easily penetrate through the fungal cell wall. HPMC was found to be the best mucoadhesive polymer as it showed the longest residence time. Moreover, a controlled drug-release effect was reported in the formulation containing xanthan gum [84].

### 3.3. Vaginal Nanofibers

Nanofibers are composed of interconnected polymeric fibers that have emerged as an appealing nanostructured system as controlled release of drugs and pharmacologically active molecules can be achieved, attributed to its porous structure that allows diffusion from the polymeric matrix [85]. In order to explore this platform, metronidazole-loaded nanofibers were developed by Tuğcu et al. with polyvinylpyrrolidone (PVP) for treatment of BV. It was reported that the mucoadhesiveness of metronidazole-loaded nanofibers improved with increasing concentration of PVP due to the intermolecular hydrogen bonding between PVP and mucin, suggesting its prolonged residence in the vagina. From in vitro release assays, more than 80% of metronidazole was released in five minutes and there was complete release within 15 min due to a reduction in diffusion distance by virtue of the large surface-to-volume ratios and small dimensions of nanofibers. Ex vivo permeability studies revealed the enhanced permeability of metronidazole through the cow vaginal mucosa from metronidazole-loaded nanofibers compared to the solution and gel systems. This increment was associated with the hydrophilic properties of PVP resulting in rapid wetting of nanofibers [86]. A similar study was performed by Nematpour and colleagues with the developed nanofiber-loaded clotrimazole for local treatment of VVC. Nanofiber was prepared using the electrospinning technique with sodium alginate and dextran to improve patient acceptability by enhancing water solubility and the clotrimazole release rate. It exhibited a higher antifungal property with lower inhibitory concentration range tested against two *Candida* sp. as compared to vaginal films. This was assured by the two-fold increase in its mucoadhesive properties attributed to the greater surface area that allowed rapid wetting and gel formation. Cell viability remained above 70% in the MTT assay demonstrating no significant cytotoxicity in human gingival fibroblast (HGF) cells was induced by clotrimazole loaded nanofiber and was considered safe at concentrations of 5, 10 and 20 μg/mL in 24 h [87].

Another study undertaken by Souza et al. formulated nanofiber using PLGA incorporated with amphotericin B (AmB) as an alternative therapy for VVC. In vitro release studies reported approximately 12.5% of AmB was released every 24 h and depleted on the eighth day, suggesting drug release for an extended period in a controlled manner with no initial or final burst release, assured by complete drug entrapment into the polymeric matrix by optimizing electrospinning conditions. Excellent in vitro and in vivo antifungal activity was observed with a lower minimum inhibitory concentration (MIC) and complete clearance of vaginal fungal burden in murine model of VVC after 72 h that was further confirmed with the absence of hyphae. This positive therapeutic outcome was attributed to direct contact with the fungal cells and controlled release of AmB from PLGA nanofiber. Histological analysis in rats revealed the normal appearance of the kidney and heart, indicating the absence of nephrotoxicity and cardiotoxicity with intravaginal application of AmB-loaded PLGA nanofibers. These findings demonstrate the potential of PLGA nanofibers as an alternative scheme for treatment of VVC that ensure patient compliance [88].

In summary, exploration of nanofiber delivery tools can be effectively investigated for effective and prolonged delivery of therapeutics, which might be by incorporating into some other scaffold (e.g., film), in the treatment of vaginal infections.

### 3.4. Vaginal Films

Polymeric films are another area explored, which undoubtedly has advanced significantly, to deliver pharmaceuticals in the treatment of vaginal ailments. The advantages of gels and solid pharmaceutical dosage forms are amalgamated in the film dosage form where versatility of polymers provides the platform to develop films of desired characteristics [89]. Application of these films has been extended to evaluate against vaginal bacterial [90], viral [89,91] and fungal infections [92].

Being BV the most commonly seen vaginal infection, several researchers explored novel approaches using this mucoadhesive polymeric platform. Recently, Abilova et al. prepared a vaginal film using chitosan and poly(2-ethyl-2-oxazoline) (POZ) polymers with ciprofloxacin for the treatment of vaginal infections. Chitosan is used in the formulation due to its mucoadhesive and antibacterial activity while POZ provides water solubility to the formulation. It was found that the film containing pure chitosan exhibits stronger mucoadhesive property than the film containing the combination of chitosan and POZ due to the electrostatic attraction between cationic chitosan and the negatively charged vaginal mucosa. In addition, POZ is a non-ionic polymer, which possesses a weak mucoadhesion property. This study shows that combination of cationic and non-ionic polymer in the formulation would greatly reduce the mucoadhesive property as the non-ionic polymer occupied and decreased the concentration of the more mucoadhesive component (chitosan) [93]. Tentor et al. also performed a study on the chitosan-based membrane for vaginal application. The metronidazole-loaded alginate-chitosan (AC) membrane demonstrated good mucoadhesion towards the vaginal tissue and no toxic effects were observed on the tissue exposed to the membrane components [94]. Both formulations from Abilova et al. and Tentor et al. were effective against *E. coli*, *S. aureus*, and *G. vaginalis*, which are the common pathogens found in BV. These studies suggested that the AC membrane and chitosan-based film are potential formulations for the management of BV [93,94]. Besides that, Jalil et al. had developed an antimicrobial vaginal film using the thiolated gellan gum and loaded with metronidazole to treat vaginal infection. The thiolated gellan gum was formed by the conjugation of the S-protected ligand 2-(2-amino ethyldisulfanyl) nicotinic acid (AMENA) and the gellan gum via amide bond formation, namely S-protected gellan gum (S-GG). To study the mucoadhesion property of the polymeric films, two types of S-GG were synthesized, which were S-GG 81 and S-GG 174. S-GG 174 contained twice the amount of AMENA than S-GG 81 and, therefore, had a higher amount of free thiol groups. The ex vivo mucoadhesion results showed that the S-GG 174 and S-GG 81 films had mucoadhesive strength of 3-fold and 1.5-fold greater than the non-thiolated gellan gum films. S-GG films formed mucoadhesive gels with prolonged residence time via the interaction of the thiol groups and the vaginal mucus. As for conventional gellan gum, the films and the mucosal surface interact via weak ionic interaction which cannot sustain for a long period [95]. Calvo et al. had performed a study on the development and characterization of the chitosan-HPMC ticonazole films for the treatment of vaginal candidiasis. The biocompatible polymers that were chosen to develop the mucoadhesive vaginal films are chitosan and HPMC. The compositions of the developed film include different concentrations of chitosan, HPMC, polyethylene glycol 400 (PEG 400), and a constant amount of ticonazole. The in vitro mucoadhesive strength of the formulated films showed no significant differences. They mentioned that previous reports found that a high swelling index may decrease the adhesive strength of a formulation. The authors explained that overhydration of the polymers can affect the adhesive strength due to the disentanglement of the polymers at the interface. Although the film with 1% *w/v* chitosan, 5% *w*/*w* PEG 400, and without HPMC showed the highest swelling ability, it is insufficient to affect the adhesiveness, resulting in similar mucoadhesive strength of all the formulations being observed [96]. Mishra et al. designed a clotrimazole mucoadhesive film based on different compositions of hydroxypropyl cellulose (HPC), sodium alginate, and different ratios of the plasticizers. This study suggested that HPC is responsible for mucoadhesion and up to a certain extent sodium alginate delays the drug release with increasing concentration. Besides that, an increase in the concentration of propylene glycol, one of the plasticizers, resulted in overhydration of the polymer and reduced the mucoadhesive strength of the film due to disentanglement at the surface of contact between the polymer and the vaginal mucosa. It was highlighted that the films were superior to the marketed Candid-V6^®^ tablets without suppressing the growth of *Lactobacillus* sp. [97].

Alternatively, Cazorla-Luna et al. developed a bilayer vaginal film based on ethylcellulose to deliver tenofovir (TFV) for the pre-exposure prophylaxis of HIV in women. The mucoadhesive property of two natural polymers, tragacanth gum (TG) and xanthan gum (XG), were being studied. Based on the results, it was observed that large amount of natural polymer provides higher quantity of the mucoadhesive functional groups to interact with the vaginal mucosa thus increasing the adhesive strength as compared to the film with a lesser amount of natural polymer. It was also found that XG containing films exhibit stronger mucoadhesive strength compared to the films with TG. The mucoadhesive time of the films containing XG was significantly longer than the films containing TG. Three human cell lines, which were the lymphoblastic cell line (MT-242), macrophage-monocyte derived cell line (THP-1) and uterine/endometrial epithelial cell line (HEC-1A), were used in the cytotoxicity study of the formulations. The materials used to develop the vaginal film including ethylcellulose, TG, XG, glycerol and tributylcitrate were tested on the cells after a 48 h incubation at room temperature. The results showed that all the materials were biocompatible and non-toxic to the three different cell lines. Overall, all the materials used to produce the TFV vaginal film were safe and non-toxic to human cells [91]. In another comparative study, two different films loaded with TNV and efavirenz were developed and tested for characteristics, pharmacokinetics profile and safety in experimental animal models. Here, the nanoparticles of the drugs were incorporated in the fabricated vaginal films, which were reported to be retained in the vaginal tissues and lavages for longer period of time, with minimal exposure of the therapeutics systemically [98].

Overall, it can be summarised that the vaginal film platform can be effectively explored in the delivery of drugs in the treatment of associated conditions of vaginal infections for improved efficacy.

### 3.5. Mucoadhesive Polymeric Approaches

The healthy vaginal mucosa consists of a multilayered stratified squamous epithelium that rests on a lamina propria. The epithelium undergoes differentiation and contains several distinct layers of the stratum basale, the superbasal layer, and the stratum corneum. The structure of the vaginal epithelium changes throughout a woman’s lifetime according to the hormonal and environmental conditions. The vaginal epitheliums of young girls before puberty are thin and only consist of the basal and parabasal layers. Upon the reproductive ages, the vaginal epithelium thickens and starts to develop a cornified layer. It becomes thinner after menopause, diminishing the glycogen storage and keratinization is occurred in the stratum corneum. The vaginal *stratum corneum* is enriched of mucins that lead to the attachment and retention of the microbes in the case of BV [99]. Mucin is the major component present in the mucus along with water, proteins, cells, and lipids to form a gel-like lining at the mucosal tissues [100]. Successful delivery of the non-mucoadhesive formulations via the vagina remains a challenge, primarily due to poor drug retention and absorption across the vaginal epithelium as a result of the vaginal self-defence mechanism [100,101]. To prolong the residence time and avoid leakage of the drug administered, mucoadhesion plays an important role in the formulation. Mucoadhesive polymers interact with the vaginal mucosa to improve the adhesion of the formulation. The stronger the mucoadhesive property of the polymer, the longer the period of the formulation remains in the vagina [82]. Therefore, a mucoadhesive polymeric approach could be employed in vaginal drug delivery to improve the adhesive property of the drug at the vaginal epithelium for better drug absorption. The following sections will discuss the various studies undertaken by researchers on the mucoadhesive polymeric approaches in vaginal drug delivery for different types of vaginal infection.

In a more advanced method of delivering drugs, Tuğcu-Demiröz incorporated benzydamide hydrochloride (BNZ) loaded liposome into the mucoadhesive gel forming a lipogel for vaginal application. In this study, BNZ was loaded into the liposome, gel, and lipogel along with mucoadhesive polymers HPMC and Carbopol^®^ 974P. The ex-vivo mucoadhesion studies were conducted by evaluating the force required to detach the formulations from the cow vaginal mucosa. As measured using the texture analyser, the mucoadhesive gels and lipogels showed notable mucoadhesive strength. The highest mucoadhesion values were observed in the HPMC K100M gels Liposome formulations showed the lowest values for work of mucoadhesion among all the formulations because no mucoadhesive polymers were present. The developed lipogel was more effective than liposomes as it exhibited approximately 5-fold stronger mucoadhesive strength. Hence, HPMC K100M was shown to be a good choice of vehicle to form lipogel attributed to its mucoadhesive property that eventually prolonged the retention time of the formulation in the vagina [80].

Bhat et al. studied the use of HPMC, NaCMC, and guar gum (GG) as the polymer in the development of bioadhesive controlled release vaginal tablets loaded with clotrimazole for the treatment of VVC. The bioadhesion study showed that the formulations with HPMC and NaCMC have stronger adhesive strength towards the mucosa than the formulations with the combination of HPMC and GG. They also found that the higher the concentration of the polymer used, the greater the mucoadhesive strength that is obtained. Hydration of the polymer and adequate concentration of HPMC are necessary to initiate the mucoadhesive process between the tissues and the polymer [102]. With a similar objective, Calvo et al. performed a study on the chitosan-HPMC ticonazole films for the treatment of vaginal candidiasis. The biocompatible polymers that were chosen to develop the mucoadhesive vaginal films are chitosan and HPMC. The compositions of the developed film include different concentrations of chitosan, HPMC, (PEG 400), and a constant amount of ticonazole. Based on the in vitro mucoadhesive studies, the formulated films showed no significant differences in the mucoadhesive strength results (*p* > 0.01). They mentioned that previous reports found that a high swelling index might decrease the adhesive strength of a formulation. The authors explained that over-hydration of the polymers can affect the adhesive strength due to the disentanglement of the polymers at the interface. Although the film with 1% *w/v* chitosan, 5% *w*/*w* PEG 400, and without HPMC showed the highest swelling ability, it is insufficient to affect the adhesiveness, resulting in similar mucoadhesive strength in all the developed formulations [96]. Alternatively, Rençber et al. used HPMC K100M and E50 to develop an in situ vaginal gel using poloxamer 407 (P407) and 188 (P188) containing clotrimazole for the treatment of vaginal candidiasis. In the mucoadhesion studies, no significant difference was found between these two types of HPMC. However, it was shown that the addition of clotrimazole to the formulation decreases the mucoadhesive property of the gel. According to the mucoadhesion values, there were no significant difference among all the prepared formulations (*p* ≥ 0.05) [82].

To prolong the retention time and control the release of TFV, Meng et al. developed TFV-encapsulated microparticles by spray-dried sodium alginate and further coated with thiolated chitosan based on the charge–charge interaction. The mucin adsorption of the chitosan-coated microparticles is increased by 10- to 20-fold as compared to that of the non-mucoadhesive sodium alginate microparticles. The mucin adsorption in both vaginal fluid simulant (VFS) and semen fluid simulant (SFS) was low (20%) when the mucin concentration is comparably low (0.1 mg/mL). The mucin adsorption increased to around 50% when the mucin and the microparticles were at the concentration of 1 mg/mL. However, when the mucin concentration was increased to 2 mg/mL or above, the percentage of mucin adsorption was decreased to about 40%. This might be attributed to the negatively charged protein present in the VFS and SFS, which can interact with the microparticles via charge–charge interaction. They competed for the microparticles surface thus reducing the interaction between microparticles surface and mucin molecules [103]. To study the in vivo safety of the developed mucoadhesive microparticles, the formulations were applied intravaginally to the female C57/BL6 mice for examinations. The tissues exposed to the double layer microparticles (DLMPs) did not show significant histological abnormalities after 7 days. Hence, the results showed that the exposure of vaginal tissues to the DLMPs caused no considerable infiltration of the immune cells [103].

The studies of mucoadhesive polymeric approach in vaginal drug delivery in this review are summarized in Table 2. In summary, it could be emphasized that the deliveries containing mucoadhesive polymers could retain the formulation at the site of application for a longer period for effective absorption of the incorporated therapeutics to control infection at the vaginal site.

### 3.6. Stimuli-Responsive Approaches

VDDS is the preferred method for local treatment of vaginal infections as it shows clear advantages over the other routes, including the avoidance of first-pass metabolism, offers stable drug concentration at the infected site, and has high contact surface area. However, the drug efficacy is greatly limited by the dilution effect of vaginal secretion and the self-cleansing action of the vagina. Thus, various in situ formulations which provide prolong residence time are developed for optimal vaginal drug delivery [104,105].

In this section, we will be focusing on polymer-containing responsive systems that undergo drastic and abrupt physiological changes in response to a stimulus or stimuli. Stimuli are classified into three categories: biological (enzymes, receptors), chemical (pH, ionic strength, solvent), or physical (temperature, magnetic, light, ultrasound). Stimuli-responsive polymers respond sharply to the environment, resulting in an alteration of the polymeric structures and transformation of macroscopic properties such as bond cleavage, degradation, and changes in energy level. These polymers have received considerable attention in the pharmaceutical and biomedical fields due to their unique properties, including a lower or higher critical solution temperature and the ability to return to their initial state after the trigger is removed (reversibility) [106,107,108,109,110]. Thus, based on the available approaches, temperature, pH, ionic, combination and other responsive systems will be discussed in the following sections.

#### 3.6.1. Thermo-Responsive Systems

To meet the challenges associated with VDDS, an increasing number of thermogelling systems have been proposed in the literature. The mechanism underlying thermogelling systems is the transition of a polymeric solution into gel in response to a change in temperature. Chitosan and poloxamer are temperature-sensitive polymers widely used in the preparation of thermo-responsive hydrogels. Numerous drug-loaded thermo-responsive hydrogels have been formulated for the treatment and prevention of vaginal diseases, as this approach offers significant potential in controlled and sustained drug delivery. The sections below will describe the various studies undertaken on thermo-responsive formulations for various vaginal diseases.

**Figure 5:**
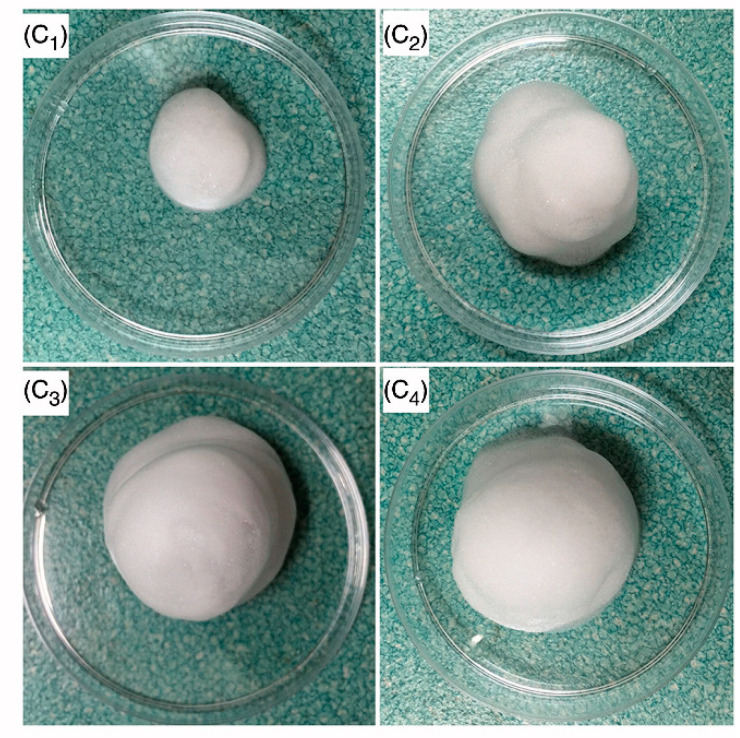
Foam properties of the optimized expansible thermal gelling foam aerosol gel (ETGFA) formulation. (**C_1_**) the pale white colour foam generated is dense and in a relatively small volume upon spurted out from the sealed container; (**C_2_**) the foam expands to a larger size with puff texture; (**C_3_**) the foam reaches the maximum expansion state within 10 min; and (**C_4_**) the foam retained the maximum expansion up to 60 min. Adapted from “Expansible Thermal Gelling Foam Aerosol for Vaginal Drug Delivery” by Mei et al. [111].

**Figure 6:**
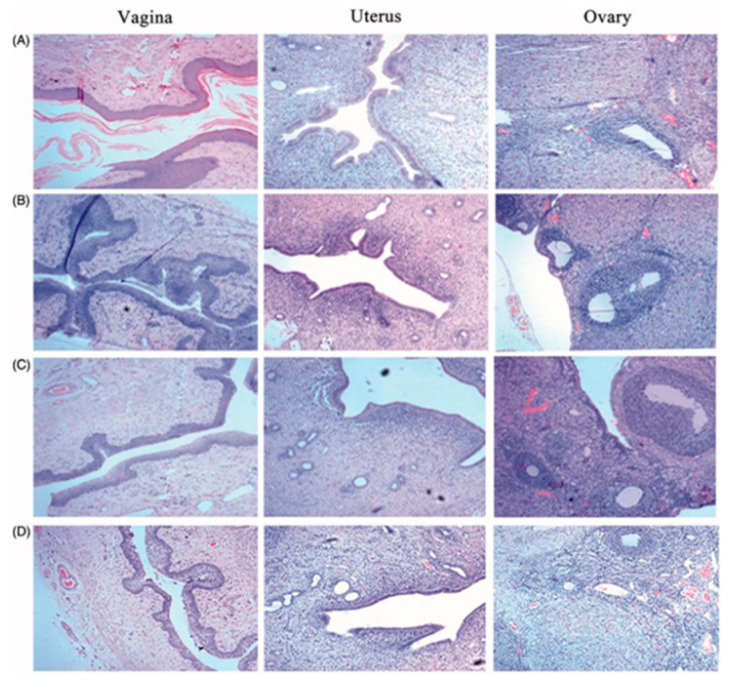
Pathological sections of vaginal, uterine and ovary tissues of Sprague Dawley (SD) rats. (Hematoxylin and Eosin (HE) staining, ×400). (**A**) for blank saline, the vaginal epithelial tissue and uterine endometrium are intact; (**B**) for blank ETGFA, hyperkeratosis of vaginal epithelial tissue and slight uterine eosinophilic infiltration are observed; (**C**) for silver nanoparticle-loaded ETGFA, hyperkeratosis of vaginal epithelial tissue, slight uterine eosinophilic infiltration and local infiltration of lymphocytes are observed; and (**D**) for silver nanoparticle solution, severe tissue irritation is observed. Reproduced with permission from “Expansible Thermal Gelling Foam Aerosol for Vaginal Drug Delivery” by Mei et al. [111].

An expansible thermal gelling foam aerosol gel (ETGFA) was designed by Mei et al. with P188, P407, and Carbopol for the treatment of vaginitis and cervical erosion. Upon administration, ETGFA foam with superior expansion height and duration time was generated with the aid of propellant (Figure 5). This improved the penetration efficiency of the formulation and avoided foam liquefaction. Moreover, addition of Carbopol increased the mucoadhesive strength of the gel. A rapid drug release within the first hour, followed by an extended release during the later stage, was reported. The instant release provided an adequate loading dose at the infectious sites. Thereby, a desirable antimicrobial effect was achieved, as the local drug concentration was comparable with the MICs of the general vaginal flora. A histopathological test with experimental Sprague Dawley rats (Figure 6) showed that the addition of silver nanoparticles into ETGFA did not stimulate additional irritation. The combination of the superiorities of foam and gel successfully improved the therapeutic efficacy of silver nanoparticles [111].

Similarly, Tuğcu-Demiröz developed a thermo-responsive hydrogel for the treatment of vaginitis. The formulation was prepared with P407 and chitosan-highly-viscous (chitosan H), a combination that possessed the highest viscosity and mucoadhesion values. The high viscosity enabled BNZ-poloxamer hydrogel to release its drug at a more sustained and controlled rate, reducing the frequency of administration In addition, incorporation of high molecular weight chitosan into the formulation had successfully improved the mucoadhesive performance of poloxamer hydrogel, as the chitosan maximised adhesion through entanglements and Van der Waals forces [112,113]. Moreover, the formulation exhibited the maximum mechanical properties by having superior hardness, compressibility, adhesiveness, cohesiveness, and elasticity. High adhesiveness, cohesiveness and elasticity are the ideal features of a vaginal gel as these permit the localisation of an antimicrobial agent at the targeted region and increase the performance of the formulation by allowing full structural recovery following administration. Appropriate compressibility and hardness are also crucial for vaginal application [112,114].

Nikhar et al. have investigated the use of thermosensitive in situ tinidazole gel in improving the treatment outcomes of BV. In this study, P407 and HPMC E100 were selected. Moderate spreadability and high mucoadhesion permitted uniform application and prolonged residence time at the infectious site, respectively. The formulation demonstrated the ideal mechanical properties of a vaginal gel. The drug-release profile of the in situ tinidazole gel was the same as the ETGFA, where there was an initial burst release followed by a controlled release. According to the zone of inhibition, the gel showed superior antimicrobial efficacy over the marketed products. A Hen’s Egg Test-Chorioallantoic membrane (HET-CAM) test revealed that the gel was slightly irritative [115].

Combating VVC, Patil et al. formulated two different vaginal gel formulations of clotrimazole, which were fabricated using different combinations of polymers, including Pluronic F127, Pluronic F68 and polycarbophil. Upon vaginal application, good spreadability of both formulations promoted sufficient coverage on the vagina, while their respective gelation temperatures allowed gelation after the temperature shift. Moreover, higher gel strength, mucoadhesive properties and viscosity were observed in formulation with higher proportion of polycarbophil. This could be attributed to the formation of more ionic and hydrogen bonds between the carboxyl groups of polycarbophil and the mucin glycoproteins that covered the mucosal epithelium. Both formulations demonstrated a more extended drug release as compared to that of the marketed preparations. Altogether, both formulations exhibited potential features that could augment the therapeutic efficacy of clotrimazole for the treatment of VVC [116]. Alternatively, an AmB-loaded gel using P407 (AmB-gel) that promotes drug permeation through the mucosa, was developed by Sosa et al. for the treatment of VVC. The formulation conferred the ability to transform into a highly viscous gel under high temperature. This could be explained by the decreased solubility of the polymeric chains as temperature rose. Eventually, micelles were formed, and viscosity of the gel increased. Furthermore, rapid degradation and highly porous structure of AmB-gel contribute to the sustained release of AmB. The high drug-retention capacity in the vaginal mucosa and the absence of AmB in the receptor chamber revealed that the formulation favoured a local effect and did not enter systemic circulation. This could be due to the presence of P407 which also functioned as a surfactant that potentiated the drug diffusion ability. Low MIC values against *C. albicans*, *C. glabrata*, and *C. parapsilosis* deduced that the formulation exhibited strong antifungal activity. This may be related to the synergistic interactions between P407 and AmB [117].

Apart from vaginal gel, another novel approach of liquid vaginal suppository was prepared by Ramandeep et al. for the treatment of VVC using P188, P407 and HPMC. It was concluded that formulation with 2.4:0.5% *w/v* of P407:P188 and 0.04% *w/v* of HPMC showed desirable gelation temperature and pH. Besides, the researchers discovered an extensive improvement in physiochemical properties of liquid suppository after the addition of mucoadhesive polymer. The superior gel strength and mucoadhesive force of the formulation were attributed to the enhanced interaction between the hydroxyl group of HPMC and the oligosaccharide chains of vaginal mucosa lining. This facilitated insertion and prevented leakage of the liquid suppository. Furthermore, a more controlled release of miconazole nitrate was observed in formulation containing HPMC which might be due to high viscosity gel that limited the penetration of dissolution fluid into the matrix. The inhibition zone diameter which corresponded with the marketed formulation indicated the promising antifungal activity of liquid suppository [118].

As a part of novel approach in drug delivery, Zhang et al. embedded auranofin (AF)-loaded NPs into a thermo-responsive hydrogel, which was formulated using chitosan and β-glycerophosphate targeting trichomoniasis. Thermal gelation was observed at high temperature followed by the formation of a cross-linked gel structure. Although AF-NP gel was less potent than plain AF, the formulation could inhibit the parasite’s growth completely. By examining the stained vaginal epithelium of female mice, the gel was well retained in the targeted region after administration. The presence of drugs in the tissue after six hours proved that the formulation was released in a sustained manner. Furthermore, no profound alteration was observed in the activity of hepatic thioredoxin reductase, an enzyme targeted by AF, and the estrus cycle in female mice. The findings illustrated that the formulation did not cause apparent systemic or topical toxicity [119].

To ensure the complete delivery of sufficient doses in the treatment of VV, Timur et al. incorporated free TFV and TFV-loaded chitosan nanoparticles into a thermogelling system. With an optimal gelation temperature, the formulation (f-TFV-CS-NPs-gel) transformed into gel in the vaginal canal. The statement was supported by the increased viscosity of f-TFV-CS-NPs-gel at higher temperature. Furthermore, TFV was released in a biphasic manner, where the initial burst release provided a loading dose for the prevention, followed by sustained drug release that helped to achieve desired concentration at the targeted site. Nonetheless, an expected increase was absent in the mucoadhesion value of f-TFV-CS-NPs-gel after the addition of the chitosan nanoparticle. The high cell viability inferred that each component was safe for vaginal application when utilised at proper proportion [120].

Another polymeric blend hydrogel was studied by Alves et al. for the treatment of vaginal mucosal inflammation and infectious disease (e.g., HPV infections). The formulation composed of P407, HPMC K4M, chitosan and curcumin in solid dispersion form (CUR-SD). The pH of close to 4.5 provided the best CUR stability, favoured the solubility and protonation of chitosan. Moreover, the hydrogel exhibited a non-Newtonian flow behaviour at elevated temperature. The erosion rate suggested that the hydrogel had prolonged residence time on the vaginal mucosal and controlled release rate for CUR. However, the addition of mucoadhesive agents did not influence the mucoadhesive properties of P407 as expected. Instead, the bio-adhesive force increased in the presence of CUR-SD, where the molecular nature of the drug enabled the establishment of hydrogen bonds with the mucin. By evaluating for oxidative stress with HeLa cells, the formulation showed the best antioxidant capacity as the good solubility and stability of CUR-SD made it more bioavailable in protecting the cells from oxidative damage [121].

Table 3 illustrates the polymer used, disease type, cell line or animal model used and outcomes of different thermo-responsive formulations. The findings of reported research discussed so far point towards effective delivery of therapeutics against vaginal infections using this thermoresponsive polymeric platform.

#### 3.6.2. pH-Responsive Systems

pH is an important parameter in VDDS as variation in vaginal pH significantly influence the performance and efficacy of a formulation. The presence of infection and seminal fluids are the common factors contributing to the rise in pH. Besides hydrogel, vaginal films and membrane are the pharmaceutical dosage forms available for vaginal application. Recent studies focused on the development of pH-responsive systems for the pre-exposure prophylaxis of HIV infection.

In this context, a TFV-based biphasic system containing a mixture of organogels and hydrogels was developed for the prophylaxis of HIV infection. The bigel was freeze-dried to increase its viscosity and to enhance the interaction between the polymer chains and vaginal mucosa. This resulted sustained drug release and greater mucoadhesion. Moreover, the high concentration of pectin created a structure with a dense polymeric frame and small pore size. This limited the amount of water trapped in the formulation, causing it to be hard and less deformable. The high degree of esterification of pectin has contributed to the superior mucoadhesive properties of bigels because of the formation of more hydrogen bonds between the carboxylic groups of pectin and the mucin glycoproteins of vaginal mucosa. The bigels experienced a faster loss of structure in a stimulated vaginal fluid (SVF)/stimulated seminal fluid (SSF) mixture than in SVF, as the acidic character of pectin made it more soluble at higher pH. This led to a rapid drug release. [122].

Moreover, Cazorla-Luna et al. have developed a vaginal film based on TFV by employing the layer-by-layer technique, which combined the properties of the two layers of polymers, chitosan citrate and methyl polymethacrylate (Eudragit^®^ S100). The synergy between the polymers produced a polyelectrolyte complex that showed improved stability and pH-dependent behaviour. The vaginal film was highly resistant and flexible due to the presence of ES100 and citric acid, which crosslinked chitosan derivatives and facilitated the mobility of the polymer chains. The crosslinkers also contributed to the low swelling capacity of the film by creating a three-dimensional structure that impeded water penetration. Additionally, the low water uptake and a high proportion of ES100 improved the mucoadhesion capacity of the formulation by increasing the interaction between chitosan and mucosa. Similarly, the drug-release behaviour was assessed in SVF and a SVF/SSF mixture. In SVF, the crosslinking chitosan and ES100 hindered water access. This decelerated drug dissolution and resulted in an extended drug release. In contrast, chitosan was the only polymer responsible for TFV release in SVF/SSF as ES100 became soluble at higher pH. This contributed to a rapid drug release. Cytotoxicity experiments deduced that citric acid and ES100 were non-toxic and demonstrated little damage to mucosal integrity [123].

**Figure 7:**
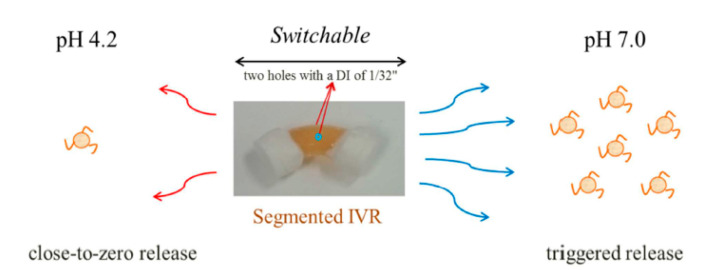
Diagram of pH-responsive supramolecular polyurethane (PU) hydrogel in reservoir-segmented intravaginal ring (IVR). The fabrication process of segmented IVR are as follows: (1) The reservoir of IVR are filled with PU hydrogel. (2) The ends are capped with custom-fabricated plastic lids and sealed with polymer resin. (3) Two holes are made for the switchable on-demand release of siRNA-loaded nanoparticles. Reproduced with permission from “Switchable On-Demand Release of a Nanocarrier from a Segmented Reservoir Type Intravaginal Ring Filled with a pH-Responsive Supramolecular Polyurethane Hydrogel” by Kim et al. [124].

To increase the delivery efficiency of small interfering RNA (siRNA), Kim et al. designed two pH-responsive switchable formulations, namely the supramolecular polyurethane (PU) hydrogel and PU membrane. In the first study, siRNA-loaded nanoparticles were encapsulated in the PU hydrogel and the final formulation was physically crosslinked within a segmented reservoir-intravaginal ring (IVR) (Figure 7). The in vitro release profiles revealed a close-to-zero release at pH 4.2 but a sustained release at pH 7.0. This was related to the pH-responsive modification in physicochemical interaction of copolymer chains. Deprotonation of 2,2-dimethylolpropionic acid at pH 7.0 created a relatively weak intermolecular hydrophobic interaction. The space between the particles enabled a rapid nanoparticles release. Moreover, the close-to-zero value of zeta potential enhanced the mucus penetration of nanoparticles. The nanoparticles and hydrogel exhibited no and low cytotoxicity towards the cell lines, respectively [124].

**Figure 8:**
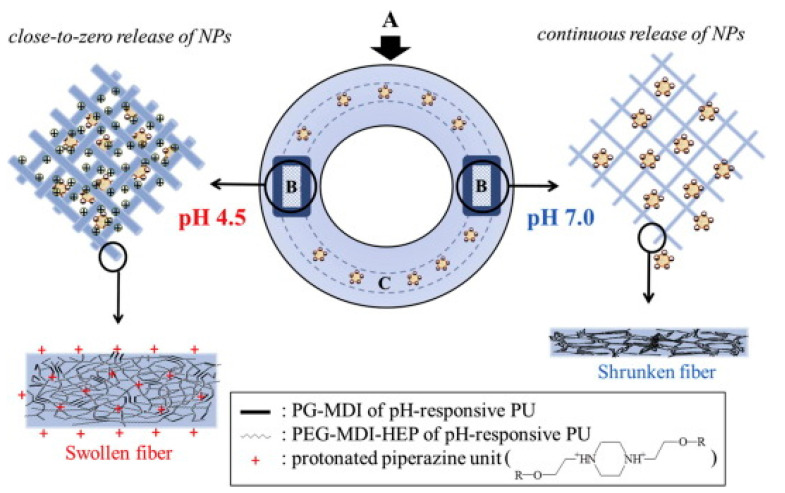
Diagram of the electro spun porous pH-responsive membrane as a “window” membrane in reservoir-intravaginal rings for controlled release of siRNA-loaded nanoparticles. (**A**) intravaginal ring; (**B**) window membrane; (**C**) reservoir. pH-responsive changes in the morphology of the membrane and electrostatic interaction between the pH-responsive membranes and anionic nanoparticles contribute to the release of nanoparticles. Adapted from “Design and Development of pH-responsive Polyurethane Membranes for Intravaginal Release of Nanomedicines” by Kim et al. [125].

Another formulation approach was made through fabricating nanofibers using electrospinning of the pH-responsive PU copolymers. The in vitro release profile of the formulation was comparable with PU hydrogel. In an acidic environment, protonation of the 1,4-Bis(2-hydroxyethyl) piperazine polymer caused swelling of electrospun fibers. Consequently, a PU membrane with smaller pore size, larger diameter and thicker cross-section was formed. Moreover, a strong attractive force was detected between the siRNA-loaded nanoparticles and PU membrane. These modifications restricted the penetration of the drug-loaded nanoparticles across the membrane, resulting in a lower drug release. The opposite findings reported in the alkaline environment caused an accelerated drug release (Figure 8). The formulation did not cause any remarkable toxicity and changes in the expression of proinflammatory cytokines [125].

Cervical mucus is a natural barrier present in the vaginal lumen that impedes the diffusion of viral particles and cells. However, the dilution and neutralization effect of biological fluids (e.g., vaginal and seminal fluids) would break down the barrier function. Sexual transmission of HIV takes place when the semen-borne virus moves through the compromised cervical mucus and penetrates the cervicovaginal epithelium. Inspired by the ability of CVM to trap HIV virions, Mahalingam et al. constructed a pH-responsive synthetic mucin-like polymer system (SMP) which functioned as a microbicide drug-delivery vehicle and an efficacious physical barrier to the transport of HIV. At elevated pH, a highly viscous gel layer with small mesh size and covalently crosslinked elastic network was formed due to the long lifetime of the crosslinked polymer. Unlike previous work, this system illustrated both shear-thinning and shear-thickening behaviour, which improved the coverage and retention of the gel layer on the vaginal tissues. Moreover, the interaction between phenylboronic acid and vaginal mucin contributed to the mucoadhesive properties of the SMP. Limited migration of HIV virions and macrophages in response to the addition of semen indicated the ability of SMP in resisting dilution and impeding the movement of cells and viral particles. Cytotoxicity evaluation of SMP showed no significant inflammatory response, loss of cell viability, and tissue integrity [126].

Table 4 demonstrates the polymer used, disease type, cell line or animal model used and outcomes of different pH responsive formulations. Similar to the thermo-responsive polymeric approach, pH-responsive polymers are effectively contribute towards superiority in drug delivery in vaginal infections, where the pH of the vagina facilitate these delivery systems.

#### 3.6.3. Ion-Responsive Systems

A stimuli-sensitive approach has been incorporated in vaginal drug delivery to overcome the limitation of conventional vaginal preparations such as poor residence time which leads to poor drug absorption. This is because it combines the advantages of the gel and solution forming the in situ gelling system. Using this gelling system, the formulations will remain in a solution state until they are administered [127,128]. Human biological fluids consist of various types of ions that can be utilized to activate the drug delivery system. As for vaginal fluid, it consists of sodium ions (Na^+^), potassium ions (K^+^), calcium ions (Ca^2+^), and chloride ions (Cl^-^) [129]. The ion-sensitive polymers transform the liquid formulation into gel form in the presence of ions. Alginate forms gel in the presences of Ca^2+^ whereas gellan gum forms gel in the presence of Na^+^, K^+^, and magnesium ions (Mg^2+^) [19]. Both gellan gum and alginate are used in vaginal in situ gel formulations to improve the gelling and bioadhesive properties of conventional vaginal gels [19,127]. However, only a limited number of studies have been reported on ion-activated VDDS.

Gupta et al. developed and optimized a bioadhesive in situ vaginal gel using chitosan and gellan gum. Chitosan act as a bioadhesive polymer and permeation enhancer while the gellan gum act as the ion-activated polymer in the formulation. Clindamycin was used as the model drug as it is used in the treatment of BV. The formulation with 1% *w/v* chitosan and 1% *w/v* gellan gum was selected for further evaluation because it formed a firm and transparent gel upon administration which indicated its good gelling capacity [127]. As shown in the results of the HET-CAM test, no irritation occurred up to 2 h (mean score 0). The mean score was increased to 0.33 after 6 h and further increased to 0.66 after 24 h of exposure [127,130]. In short, this study proved that the combination of chitosan and gellan gum is able to form an in situ vaginal gel which has desirable bioadhesion and retention properties and most importantly is a non-irritant to the vaginal mucosa [127].

In another study undertaken by Patel et al., gellan gum was chosen as the ion-activated gelling polymer whereas the bioadhesive polymer used was HPMC. The in situ activated gel was developed to deliver clindamycin through the vaginal route for BV. As shown in the mucoadhesion studies, the formulation containing both HPMC and gellan gum exhibited the strongest mucoadhesive strength. It was found that using either HPMC or gellan gum alone was unable to form a desirable mucoadhesive in situ gelling system. This is because the HPMC and gellan gum play major roles as a mucoadhesion polymer and ion-activated gelling agent in the formulation, respectively [130]. The HET-CAM test results were similar to the test results of Gupta et al. This indicated that the chitosan/gellan gum based in situ gel was a non-irritant to mild irritant. Thus, it was predicted to be well tolerated by the vaginal mucosa [127,130]. Due to a lack of in vivo cytotoxicity studies, the possible adverse event of the ion-responsive in situ vaginal gel is unknown.

Besides that, gellan gum had also been used by Harish et al. to develop an ion-responsive drug delivery system for the local effect of secnidazole for the treatment of vaginal trichomoniasis. In the formulation, NaCMC was used to prolong the release of the drug. Calcium chloride and sodium citrate were added into the formulations to provide free ions to further activate the gelation process of gellan gum after administration. The optimal concentration of calcium chloride and sodium citrate to achieve the satisfactory effect of 0.5% *w/v* gellan gum were 0.05% *w/v* and 0.17% *w*/*v*, respectively. As the amount of gellan gum and NaCMC increased in the formulation, the viscosity, gelling capacity, and the gelling strength of the formulations was increased [131].

Table 5 summarizes the studies of ion-responsive polymeric approach in vaginal drug delivery selected for this review. Although the number of studies is low, it can be inferred that the use of ion-responsive polymers in the delivery of drugs in the vaginal infection would provide an important platform to circumvent the issues related to conventional treatments.

#### 3.6.4. Multi-Stimuli Responsive Systems

In the last decade, novel multi-stimuli responsive systems that respond to a combination of two or more environmental stimuli have been developed for biomedical applications. These systems open new prospects for ‘smart’ vaginal delivery. The combined effect of multiple stimuli provides the ability to fine-tune the response to each stimulus independently. Moreover, the drug-release profile may be regulated to achieve controlled release or to preserve the primary drug until the intended target is reached [107,132].

Multi-stimuli responsive systems can be developed by utilising two or more polymers that are responsive to different stimuli. This approach not only allows the reduction of concentration required of each polymer but also provides synergistic effects due to the responsiveness to multiple stimulations [107,132]. For instance, the use of gellan gum in combination with Pluronic results in an augmented response in terms of gelling sensitivity [133,134]. Other than utilising two polymers that respond to different stimuli, a more recent technique is by combining two or more stimuli-responsive moieties within a single polymeric system. The rationale is to merge different monomers with distinct stimuli-responsive behaviours to form a copolymer that has dual- or multi-responsive properties [135,136,137]. One of the examples of dual temperature/pH-responsive copolymers is chitosan-graft-poly(*N*-isopropylacrylamide). This copolymer can be synthesised by joining chitosan (a pH-responsive polymer) with N-isopropylacrylamide (pNIPAAm, a thermo-responsive polymer) via chemical linkages, thus generating a copolymer that responds to both stimuli simultaneously. By changing the composition of the two moieties within a copolymer, drug-release kinetics can be modified [138].

In this context, Yenkar et al. formulated a clindamycin-loaded in situ gel using a combination of temperature- and ion-activated gelling polymers, PF-127/68 and gellan gum respectively [133]. Gellan gum contributed to the conversion into a gel in the presence of divalent cations in the SVF and led to a sudden increase in viscosity. The use of carrageenan, in combination with Pluronic and gellan gum, had improved the mucoadhesive properties by slightly prolonging vaginal residence time of in situ clindamycin gels. This may be attributed to both the bio-adhesive property of carrageenan itself and its ability to slow down the erosion of in situ clindamycin gels [139]. The in situ clindamycin gel offered sustained drug release for up to nine hours compared to conventional 2% clindamycin hydrochloride cream. The results of this study have demonstrated that the combination of Pluronic, gellan gum, and carrageenan is safe and may be useful for sustained drug delivery for vaginal application [133]. Furthermore, Lin et al. designed a dual pH- and temperature-responsive sodium alginate (SA)/pNIPAAm hydrogel loaded with antibiotic oxytetracycline (OTC) for controlled drug release [140]. With increasing pH, the carboxyl groups in SA and acrylic acid (AA) moieties are gradually ionised and cause an increase in the electrostatic repulsion, thus raising the swelling ratio to the highest value at pH 10.96 [141]. The temperature sensitivity in the swelling behaviour of SA/pNIPAAm hydrogel is inherited from the individual polymer. An initial slow release of OTC during the first 24 h, followed by a constant release for the next 48 h, was observed which maintain the concentrations of OTC within the therapeutic window. A reduction in the viability of gram-positive bacteria *E. coli* along with an increase in the concentration of OTC-loaded SA/pNIPAAm hydrogel from 1 to 100 µg.mL^−1^ indicated the dose-dependent antimicrobial activity of OTC. According to the cytotoxicity assay, the OTC-loaded SA/pNIPAAm hydrogel showed high cell viability, demonstrating the potential of SA/pNIPAAM hydrogel as a biocompatible carrier for controlled release of OTC [140].

**Figure 9:**
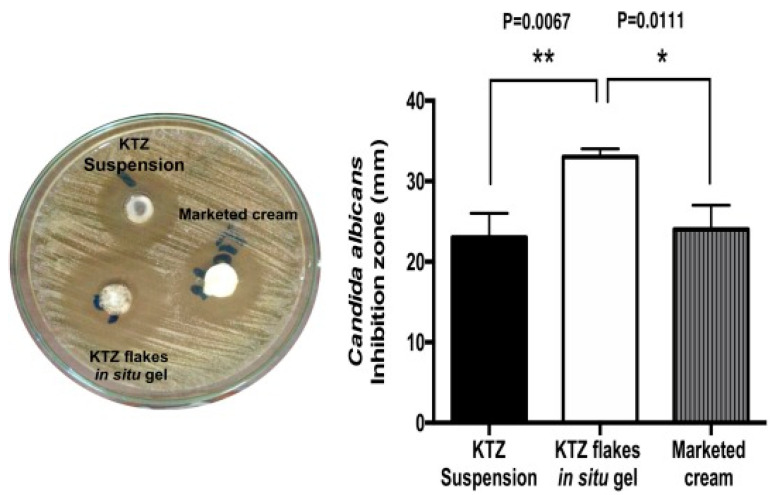
Zone of inhibition (mm) for ketoconazole suspension, marketed vaginal cream (terconazole) and ketoconazole flakes-loaded in situ gel. Adapted from “Efficacy of ketoconazole gel-flakes in treatment of vaginal candidiasis: formulation, in vitro and clinical evaluation” by Abd Ellah et al. [143].

One of the novel approaches to increase the water solubility of ketoconazole (KTZ) is by forming KTZ/β-cyclodextrin (KTZ/β-CD) inclusion complex. The encapsulation of KTZ/β-CD into gel-flakes made up of chitosan and gellan gum increased the penetration ability of KTZ to reach the submucosal layers as a result of the thin thread-like and polygonal structures of the developed gel flakes. The loading of developed KTZ gel-flakes into an in situ thermo-reversible gel of PF-127 ensured a rapid gel formation over the vaginal mucosa to prevent leakage. Slow sustained release for six hours after administration was noted because of the large swelling capacity of gellan gum and chitosan, which created pores in between the polymer matrix to aid in KTZ diffusion [142]. Furthermore, the antifungal efficacy of KTZ flakes-loaded in situ gel against *C. albicans* was compared with a KTZ suspension and a marketed terconazole vaginal cream (0.8% Gynoconazol^®^ cream). It was demonstrated that the KTZ flake-loaded in situ gel had the best antifungal efficacy among the three formulations based on the largest inhibition zones obtained (Figure 9). The findings of this study demonstrated that flake-loaded in situ gel is a potential novel vehicle for VVC treatment [143].

On the other hand, the clinical use of AmB is limited because of its toxic side effects and low aqueous solubility [144,145]. In this context, Kim et al. formulated a thermosensitive in situ vaginal gel using Pluronic-based triblock copolymer derivative (MBCP-2) loaded with AmB which could selectively degrade under acidic vaginal conditions [146]. Similar to the previous study, the incorporation of AmB as an inclusion complex with hydroxypropyl-γ-cyclodextrin (HPγCD) increased its aqueous solubility [143,145]. It was demonstrated that the AmB-HPγCD complex-loaded MBCP-2 gel underwent a sol-to-gel transition at body temperature (37 °C) and exhibited a pH-dependent degradation. A constant release for three days was observed at pH 5.0 and it was consistent with the pH-dependent gel degradation pattern. This ensured that the AmB-HPγCD complex-loaded MBCP-2 gel formed in situ in the vagina would degrade at acidic vaginal pH, releasing the loaded drugs [147]. The presence of HPγCD and MBCP-2 may decrease the cytotoxic effects of AmB as higher cell viabilities were demonstrated in the AmB-HPγCD complex and AmB-HPγCD complex-loaded MBCP-2 gel compared to free AmB. Repetitive vaginal administrations of MBCP-2 copolymer solution showed no signs of inflammation or tissue necrosis in the vaginal mucosa of female mice [146].

Moreover, Cheaburu-Yilmaz et al. formulated and optimised chitosan-graft-PNIPAAm/polyvinyl alcohol (CS-g-PNIPAAm/PVA) hydrogels loaded with voriconazole for mucosal delivery [138]. CS-g-PNIPAAm grafted copolymer was utilised due to its pH and temperature sensitivities while PVA enhanced the viscosity of the hydrogels. CS-g-PNIPAAm/PVA was used in a 75/25 *v/v* % ratio because of its non-Newtonian flow property, high adhesion, as well as low hardness and compressibility. A sol-to-gel transition temperature at 38 to 44 °C within two minutes validated the temperature-dependent behaviour of the CS-g-PNIPAAm/PVA 75/25 hydrogel. A pH-dependent swelling ability was also noted. Additionally, the timing of voriconazole loading into CS-g-PNIPAAm/PVA 75/25 hydrogel was shown to affect the drug release. Instead of adding voriconazole after hydrogel preparation, the loading of voriconazole during hydrogel preparation revealed a slower drug release pattern. It is hypothesised that the latter preparation method enhanced the interaction between the hydrogel matrix and the drug, providing sustained drug release properties. Kinetic analysis of release data showed that CS-g-PNIPAAm/PVA 75/25 hydrogel followed a Fickian diffusion mechanism. Thus, CS-g-PNIPAAm/PVA 75/25 can be used for drug-delivery systems intended for low-dose delivery such as semi-solid dosage forms for topical applications. CS-g-PNIPAAm/PVA 75/25 hydrogel loaded with voriconazole showed no cytotoxicity on the two different cell lines. Moreover, CS-g-PNIPAAm/PVA 75/25 hydrogel loaded with voriconazole demonstrated similar cell viabilities as that of free voriconazole, which confirmed that the CS-g-PNIPAAm/PVA hydrogel is a safe carrier for voriconazole [138].

**Figure 10:**
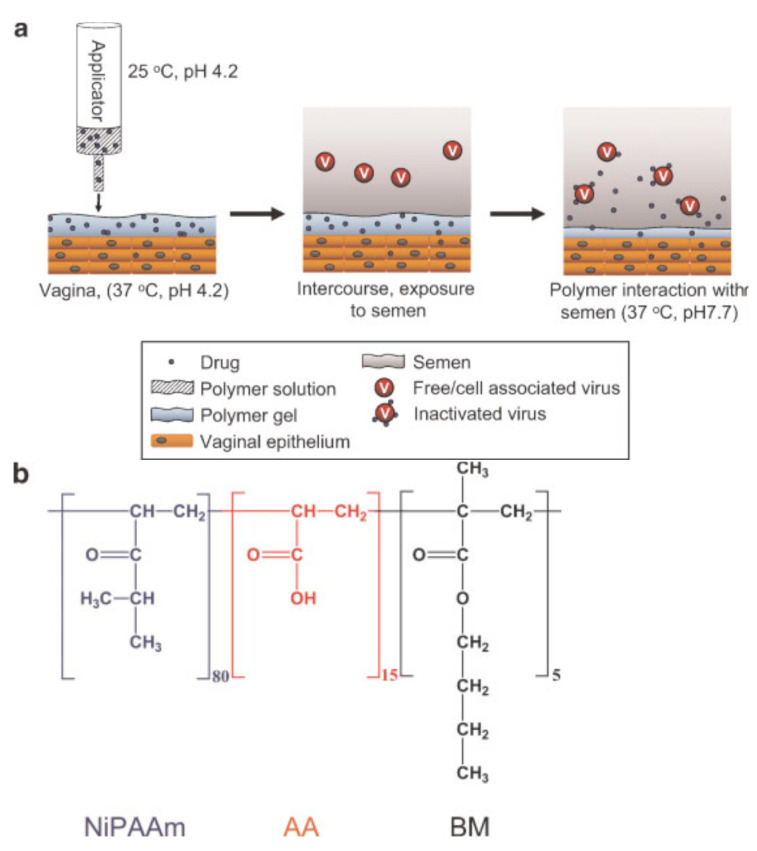
(**a**) Schematic of the expected behaviour of the polymer in response to the temperature and pH stimuli. The viscosity of the polymer solution is expected to increase due to gelation with the increase in temperature during application, thus leading to decrease in diffusion coefficient of the entrapped drugs. Upon exposure to semen, the gel loses its mechanical properties and transitions to a liquid again, which in turn will increase the diffusion coefficient of the drug drastically and promote rapid drug release along with the polymer and subsequent inactivation of virus in the semen. (**b**) Linear terpolymer of N-isopropyl acrylamide (NiPAAm), acrylic acid (AA), and butyl methacrylate (BM) synthesized with a feed ratio of 80:15:5, respectively. NiPAAm is the thermosensitive component while AA is the pH sensitive component. Adapted from “Temperature and pH sensitive hydrogels: an approach towards smart semen-triggered vaginal microbicidal vehicles” by Gupta et al. [150].

Gupta et al. designed a dual temperature- and pH-sensitive hydrogel for semen-triggered drug release using poly(NiPAAm-co-BM-co-AA) terpolymer. The hydrogel rapidly eroded upon contact with SFS (Figure 10). This is because the AA moieties of the terpolymer are protonated at vaginal pH, while at seminal pH the AA moieties ionise and become negatively charged. The repulsion between the charged polymer chains at seminal pH and the osmotic pressure cause the gel to return to its original state as a solution, resulting in a burst release of the loaded drug [148]. This helps to prevent unnecessary drug exposure to the vagina before encountering the semen, which potentially carry a high HIV viral load. It was noted that the in vitro cytotoxicity of poly(NiPAAm-co-BM-co-AA) terpolymer at 70 mg/mL was comparable to the commercial concentrations of Carbopol 974P in vaginal products, suggesting that poly(NiPAAm-co-BM-co-AA) is safe and biocompatible for vaginal applications [149,150].

Utilising temperature and enzyme as the stimuli combination, Ilomuanya et al. developed a HA/palm oil-based organogel loaded with maraviroc (MRV), an antiretroviral drug, for pre-exposure prophylaxis of HIV [151]. Since MRV is hydrophobic in nature, the utilisation of organogel as a carrier helps to optimise the release of MRV as opposed to a hydrogel, which is a hydrophilic matrix [152,153]. The percentage release of MRV in the presence of hyaluronidase was 2.5-fold higher than that in the absence of hyaluronidase. This is because hyaluronidase initiates the enzymatic breakdown of free carboxylic acid groups of glucuronic units in HA [154,155]. Based on the in vitro efficacy test, a reduced HIV infectivity was observed in cells treated with the optimised organogel compared to the untreated cells, demonstrating that the organogel could release MRV, which was then delivered and kept in sufficient concentrations inside the cells to avoid HIV infectivity. The optimised organogel was proven to preserve the viability of *Lactobacillus crispatus*. It is a desirable feature for all vaginal microbicides because the acidic environment of the vagina is maintained by *Lactobacilli*, which provides a natural defence mechanism and lowers the risk of HIV infection [44,156]. Moreover, the in vitro cytotoxicity assay revealed that the optimised organogel was non-cytotoxic, in contrast to nonoxynol-9 gel. The findings of this study proposed that HA/palm oil-based organogel could be a safe and mucoadhesive vehicle for vaginal microbicides [151].

**Figure 11:**
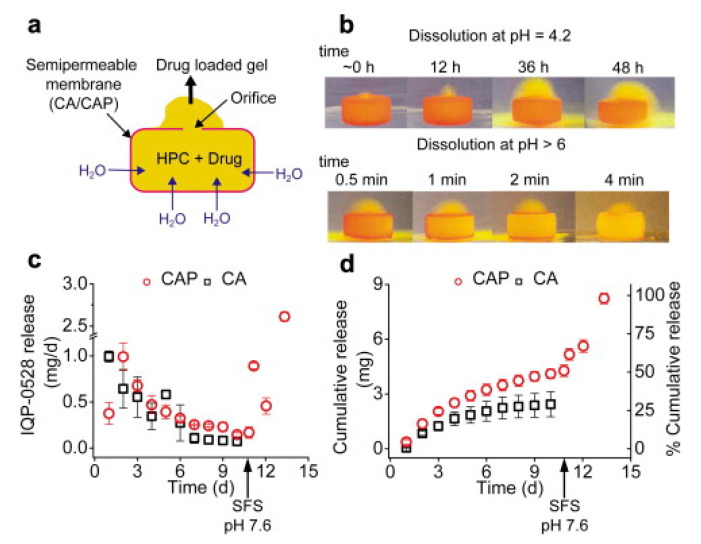
In vitro IQP-0528 release from osmotic pump tablet (OPT). (**a**) Diagram showing working of an OPT. Water is driven through the semipermeable membrane (cellulose acetate (CA)/cellulose acetate phthalate (CAP)) into the core (HPC + Drug) due to osmotic pressure difference, causing polymer swelling and extrusion of a drug loaded gel though the orifice. (**b**) Photograph showing a dye loaded CAP-OPT in release media at pH 4.2 (upper panel) and >6 (lower panel). Extrusion of a dye-loaded gel can be seen within the first hour. Upon increase in pH > 6, the CAP coating was instantaneously seen to dissolve leaving a gelled core. (**c**) Daily and (**d**) cumulative release of IQP-0528 from CAP- and CA-OPT in 25 mM acetate buffer pH 4.2 (simulated vaginal conditions) followed by mimicking pH increase by changing release media to seminal fluid simulant (SFS) pH 7.6 (N = 3; Mean ± SD). Adapted from “Osmotic pump tablets for delivery of antiretrovirals to the vaginal mucosa” by Rastogi et al. [157].

In contrast, Rastogi et al. developed osmotic pump tablets for vaginal delivery of IQP-0528, an antiretroviral drug, for HIV prevention [157]. Upon contact with SVF (pH 4.2), the HPC core drew water from the surroundings, causing the polymer to swell and extrude through the orifice into the vaginal canal (Figure 11). The in vitro release study demonstrated that the gel at this time showed slow consistent release of IQP-0528. Upon contact with SSF (pH 7.6), the semi-permeable coating of the osmotic pump tablet, which was made of cellulose acetate phthalate (CAP), was observed to dissolve immediately, resulting in a burst release of IQP-0528. The pH-sensitive burst release may be attributed to CAP, an enteric coating polymer, which is soluble at seminal pH (around pH 6 to 8) but not at the vaginal pH (around pH 4) [158]. Thus, the osmotic pump tablet was proven to be a pH-sensitive drug delivery system where it consistently released IQP-0528 at vaginal pH, followed by a burst release triggered by a coitally-associated pH increase. Moreover, the advantage of an osmotic pump tablet over a conventional vaginal tablet was investigated by administering a single vaginal dose of the respective tablet in a sheep. The IQP-0528 osmotic pump tablet was revealed to obtain stable drug concentrations for up to 10 days in the vaginal mucosa and fluid, while conventional vaginal tablets had complete drug release within 48 h, indicating that osmotic pump tablets could be novel dosage forms for sustained drug release of vaginal microbicides [157].

Furthermore, Chen et al. developed a dual temperature- and pH-sensitive in situ liposome gel (lipogel) loaded with arctigenin for vaginal administration [159]. Methoxy PEG 2000-hydrazone-cholesteryl hemisuccinate (mPEG-Hz-CHEMS) polymer was engineered as pH-sensitive liposomes which would degrade selectively at acidic pH. P407 and P188 (thermosensitive gelling polymers) were then dissolved in the pH-sensitive liposomes to form a dual-sensitive lipogel. The dual-sensitive lipogel also exhibited a pH-dependent erosion, where it presented a constant release at acidic condition (pH 5.0) for three days while a negligible release at neutral and alkaline conditions (pH 7.4 and 9.0). In short, the dual-sensitive lipogel will undergo temperature-induced sol-to-gel transition, followed by selective drug release at acidic conditions due to mPEG-Hz-CHEMS polymer degradation that is primarily caused by cleavage of acid-labile acetal bonds [160,161]. The dual-sensitive lipogel showed higher cell viability than that of free arctigenin. This study demonstrated that the combination of a thermosensitive gel and pH-sensitive liposome using poloxamers and mPEG-Hz-CHEMS as polymers had a potential to reduce the toxicity of arctigenin during vaginal delivery while providing constant drug release [159].

Table 6 summarises intravaginal formulations sensitive to multiple stimuli that have been explored for vaginal applications to date. Therefore, a combination of polymers responsive to different stimuli might be more useful for delivering drugs effectively within the vaginal environment with a superior research outcome.

#### 3.6.5. Other Responsive Systems

Liquid crystal precursor systems have been highlighted because of their low viscosity that ease vaginal administration and undergo phase transition to form liquid crystalline matrices in situ upon incorporation of water from vaginal mucus. The use of *Syngonanthus nitens* extract (SNE) in treating vaginal infections caused by *C. krusei* was studied. The transparent liquid system (TLS) exhibited a higher viscosity and a modification in its structure where it displayed a hexagonal structure (striae) and no longer possessed the characteristics of microemulsion upon dilution with artificial vaginal mucus (AVM), promoting mucoadhesion and sustained drug delivery. Similar results were obtained with the TLS loaded with extract, which showed that the loading of extract did not affect the precursor profile and flow behaviour of the liquid crystals, suggesting its feasibility for vaginal drug delivery. A decrease in MIC values was reported with SNE-loaded into the drug-delivery system. SNE-loaded in liquid crystal precursor mucoadhesive system showed prophylactic profile and inhibitory action against *C. kursei* strain, attributed to the aggregation of surfactants in the presence of water that triggered the formation of semisolid crystalline structures upon contact with vaginal mucus and the increased cell permeability triggered by oleic acid that promote the uptake of SNE [162]. Similar results were obtained when studied against *C. albicans* [163].

Phytantriol based in situ liquid gels (PILGs) designed by Jie et al. demonstrated an excellent stability and fluidity at physiological temperature with a viscosity that remained low with increasing temperature up to 42 °C. PILGs was transformed to LC gels with a small amount of vaginal fluid in a short period of time, suggesting ease of vaginal application. Superior in vivo retention of cubic LC gels and its sustained liberation of sinomenine hydrochloride was achieved by its high viscosity that resisted the vaginal cleansing action by strong adhesion onto the vaginal mucosa. An in vivo irritation study was conducted on PILGs illustrated little irritation with mild capillary hyperaemia and inflammatory cell infiltration that remained acceptable for vaginal topical preparation [164].

Araújo and colleagues designed a mucoadhesive in situ gelling liquid crystalline precursor system with the aqueous phase composed of P407. Chemical interaction of poloxamer ethoxylated groups with the mucins through hydrogen bonding upon contact with the aqueous environment, forming a more rigid system that was significantly more mucoadhesive. Sustained release property of this system was due to the interaction of hypericin (HYP) and cholesterol and a subsequent aggregation, resulting in delayed release of HYP. Furthermore, the formation of a depot system with the mucoadhesive components which allowed a gradual release of HYP. Cytotoxicity assay performed on the liquid crystal precursor system incorporated with HYP demonstrated a decreased in cytotoxicity upon dilution with vaginal simulated fluid with cell viability above 80% [165].

Table 7 represents the discussed liquid crystalline precursor systems designed for vaginal drug delivery. Thus, researchers are engaged themselves to bring up novel approaches in the delivery of therapeutics within the vaginal condition in order to treat associated infections.

## 4. Progresses of Advanced Drug Delivery in Clinical Research

Advancement in laboratory research towards improved delivery of therapeutics in the treatment of vaginal infection has led towards overcoming the barrier of the laboratory for multiple studies, which are at different stages of human trials. For example, a phase 3, double-blinded, placebo-controlled study was conducted to confirm the efficacy and safety of astodrimer 1% gel for treatment of BV and prevent recurrence. It is a dendrimer-based intravaginal mucoadhesive gel. The treatment was given at a dose of 5 g vaginally once daily for 7 days. A clinical cure was the primary endpoint of this study, which was defined as the absence of BV vaginal discharge, the presence of less than 20% clue cells, and a negative whiff test at days 9 to 12 of the study. The results showed that astodrimer was superior for the women in resolving BV symptoms at 9 to 12 days as compared to the placebo. The clinical cure rates at days 21 to 30 were similar but the difference between the astodrimer group and placebo group was not statistically significant. The overall incidence of adverse events and adverse events possibly associated to the treatment were slightly higher but insignificant in astodrimer group than that of the placebo group. Nevertheless, the reported adverse events were mostly mild to moderate, tolerable and self-limiting. The results of this trial supported the assertion that astodrimer 1% gel is safe and effective for the treatment of BV in women [166]. Curatek Pharmaceuticals completed another multi-centre phase 3 study of terconazole gel for the treatment of vaginal infection. Two hundred and twelve patients were recruited in this study to evaluate the safety and efficacy of the formulated gel formulation (NCT02308046) [167].

A phase 1, randomized, double-blind, placebo-controlled study had been performed to evaluate the safety and acceptability of SPL7013 Gel (VivaGel^®^) in sexually active women. VivaGel^®^, is a dendrimer-based intravaginal microbicide gel currently being developed to prevent STIs and as a treatment for BV. The VivaGel is a SPL 7013 formulated water-based gel consists of methylparaben, propylparaben, propylene glycol, ethylene diamine tetraacetic acid (EDTA), glycerin, purified water, sodium hydroxide and the mucoadhesive polymer, Carbopol 971P. Another two gels that were used in this study were the VivaGel placebo, which contains no SPL 7013, and the HEC gel. A total of 61 women participated in this study and randomly assigned to the VivaGel group (n = 22), VivaGel placebo group (n = 21) and HEC gel group (n = 18). The participants were treated with 3.5 g of the gel according to their group assigned every 12 h for 14 days. Analysis using the pair-wise comparison showed that the incident rate of genitourinary adverse events in the VivaGel group was significantly higher than the incident rate in the HEC gel group. Nugent score assessment showed that minor shifts of vaginal microflora were observed after the exposure to VivaGel and VivaGel placebo but it has no impact on BV. Although VivaGel had a higher incident rate of low grade genitourinary adverse events as compared to VivaGel placebo and HEC gel, the author concluded that it was safe and tolerable in sexually active women [168].

On the other hand, a triple-blind clinical trial was performed to compare the efficacy of metronidazole gel with sucrose gel. Sexually active premenopausal women suffering with BV were enrolled in the treatment. Subjects were randomly divided into two groups and treated with sucrose/metronidazolegel for 14 days. Results indicated no significance difference in the effectiveness of both the gels. This trial opens the new possibility of BV treatment compared to conventional treatment [169]. Similarly, the safety and efficacy of boric acid-based vaginal gel (TOL-463) was investigated for the treatment of BV with enhanced antibiofilm activity. In a phase II clinical trial women were randomly assigned to TOL-463 vaginal gel or insert for 7 days of treatment. Data interpretation revealed that both products tolerated well without any safety issue. Further studies needed to check the recurrence rate [170].

Currently, most of the marketed mucoadhesive vaginal gels such as Advantage-S^®^, Conceptrol^®^, Gynol II^®^, K-Y^®^, and Carraguard are indicated for contraceptive purposes. As for the treatment of BV, Metrogel Vaginal^®^ is found in the market with 0.75% metronidazole as the active drug and Carbopol^®^ 974P as the gelling polymer. Besides that, Replens^®^ gel is one of the first marketed vaginal gels as a vaginal moisturizer which consists of Polycarbophil and Carbopol^®^ 974P. It has strong mucoadhesive property where it retained in the vagina mucosa for 3 to 4 days after application [171]. As demonstrated by the marketed formulations, mucoadhesive vaginal preparations were proved to be safe and reliable for various purposes.

Metronidazole (MTZ) vaginal gel is a standard treatment of BV. However, the commercially available products consist of certain limitations (e.g., drug drainage) that discourage patients from using the vaginal applications. Furthermore, continuing treatment with MTZ is not suggested as it may result in potential toxicity and limit the growth of beneficial flora. Thus, in situ MTZ vaginal gel has been proposed to resolve these issues. A pilot randomized controlled trial (RCT) (NCT02365389) was conducted by Shaaban et al. to analyse the efficacy of the use of once-daily in situ MTZ vaginal gel and twice-daily conventional MTZ vaginal gel in the treatment of BV. Cure rates of 74.5% and 66.7% illustrated by the once-daily in situ MTZ gel after 1 week and 4 week from the start of the treatment compared to conventional gel with cure rate of 63% and 40% after 1 and 4th week of treatment. Results revealed that the in situ formulation was more efficacious than the twice-daily conventional MTZ gel. Additionally, this showed that the in situ MTZ gel provided a more persistent cure of BV. These findings may be attributed to the mucoadhesive properties and solution-gel transition ability of the thermo-responsive formulation, which ensured prolonged contact of the gel on the vaginal surface. Both therapies caused nearly the same minor side effects [172].

Recently, a newly developed 1.3% MTZ vaginal gel (MVG) has been approved as the adjunct therapy of BV because it was claimed as a formulation that could shorten the treatment course and provide similar efficacy as 0.75% MVG. Therefore, Chavoustie et al. have performed a phase 2 RCT (NCT01055106) to evaluate the efficacy and safety of MVG 1.3% once daily for one, three, or five days versus MVG 0.75% once daily for five days in the treatment of BV. In general, higher biological, clinical, and therapeutic cure rates were observed among MVG 1.3% groups. Furthermore, MVG 1.3% groups demonstrated a lower median time to resolution of symptoms (five days) than MVG 0.75% group (six days). Subjects receiving MVG 1.3% once daily for five days also possessed the lowest recurrence rate. Altogether, the outcomes implied a dose–response relationship, where the most effective regimens offered the highest concentration of MTZ. Moreover, the majority of the adverse effects reported in the four treatment groups were mild or moderate in intensity, indicating that all the formulations were well-tolerated by the participants. Since MVG 1.3% applied once daily for one, three or five days illustrated similar efficacy and safety as MVG 0.75% applied once daily for five days, MVG 1.3% could be an alternative to MVG 0.75% [173].

KTZ is one of the antifungal agents used for the treatment of VVC. However, poor water solubility and short retention time of the drug within the vagina limit its use via the vaginal route. To address these issues, a KTZ flake-loaded in situ gel was formulated by Abd Ellah et al. recently. A non-inferiority trial (Assiut University under number 17100413) was performed by the same authors to compare the antifungal efficacy between the KTZ flake-loaded in situ gel and a marketed terconazole vaginal cream (0.8% Gynoconazol^®^ cream). Patients were randomly assigned to one of the two groups. Group 1 patients received KTZ flake-loaded in situ gel (50 mg) once daily for three days while Group 2 patients (control group) received Gynoconazol^®^ cream (80 mg) once daily for three days. After one week of treatment, the cure rates for KTZ flake-loaded in situ gel and Gynoconazol^®^ cream were 88% and 82%, respectively. The findings concluded that the KTZ flake-loaded in situ gel was more superior than Gynoconazol^®^ cream in *Candida* eradication. This may be attributed to the enhanced aqueous solubility of KTZ as well as the thermo-sensitivity and mucoadhesive properties of KTZ flake-loaded in situ gel, which enhanced spreading of the drug and prolonged vaginal residence time. No complications or side effects were reported in both groups. Thus, KTZ flake-loaded in situ gel could be a potential treatment alternative for VVC [143].

## 5. Expert Opinion

We discussed earlier the exploration of nanostructured delivery systems in the pharmaceutical laboratory for medical purposes. Simultaneously, these deliveries have gained considerable interest in vaginal drug delivery owing to their capability to deliver drugs in a sustained manner, which is the prerequisite for superiority in the topical treatment of vaginal infections. Application to vaginal environment always faces the issues of leaking due to gravity, this a major challenge in designing the nanostructured system, which is having a mucin interaction upon vaginal administration and the self-cleansing mechanism of vagina. Moreover, variation in the physicochemical characteristics including the composition, size and surface potential results in different performance of nanocarriers. Therefore, various nanostructured systems were designed via different techniques and tailored with the choice of excipients according to the desired activities. Thus, surface modification of the nanocarriers using mucoadhesive polymers and fabricated with specific surface potential are the commonly used approaches to enhance interaction with the negatively charged mucin. In approaches to enhance the efficacy of the nanocarriers, sometimes these are entrapped in another delivery system for prolonged and controlled release of the drug from the formulation. For example, nanoparticular delivery has been shown to improve therapeutic efficacy when it was incorporated into the vaginal films [98]. Thus, a nanocarrier approach of drug delivery in the vaginal infections could be improved through incorporation into another vehicle, which will ease the delivery and retain within the site of application for prolonged release of therapeutics.

As discussed, mucoadhesion is an important process for the formulations to adhere to the vaginal mucosa. The major advantages of incorporating mucoadhesive polymers in vaginal drug delivery are to in increase the residence time, increase drug absorption, and reduce the frequency of administration. Besides vaginal infections, the current system is used for various therapeutic purposes including vaginal lubrication, infertility, contraception as well as labour inducement [171]. Mucoadhesive polymers have been used in vaginal drug delivery for various types of dosage forms such as vaginal films, vaginal tablets, vaginal gels, liposomes, microparticles, etc. Based on the literature reviewed previously, chitosan and HPMC are the mucoadhesive polymers that have been studied most extensively by researchers in the management of vaginal infections. It has been employed in drug-delivery systems attributed to its mucoadhesive, controlled release, and permeation-enhancing properties [174]. It is a biocompatible, safe, and biodegradable polymer that can be used together with other synthetic polymers such as HPMC, sodium alginate, and carbopol to improve the drug delivery system [80,96,174]. Among all the dosage forms mentioned in this review, vaginal gels contain high amount of water allowing it to apply and distribute well in the vagina, thus having better patient compliance compared to other vaginal preparations. Despite all the advantages of mucoadhesive formulations, their uses in vaginal drug delivery have been limited due to the broad inter-individual variability affecting some physiological factors. The vaginal secretions among individuals vary according to their age and different vaginal conditions (e.g., menstruation) [171]. Hence, the various conditions of the vagina may be studied further in the future in order to develop a better mucoadhesive formulation that suits each individual with different concerns. The development and aspects of mucoadhesive formulations reviewed in this article are promising for the future development of therapeutic formulations with mucoadhesive property. Researchers are also encouraged to explore more compounds with mucoadhesive property that can contribute to the drug delivery system.

The polymers discussed in the previous section to enhance the mucoadhesive property contribute towards enhanced viscosity or even forming gel in the formulation. Thus, trends are encouraged to use stimuli-responsive polymers, which will allow the formulation to be in sol-stage at room temperature, and when applied, will convert to gel at the application environment. In this context, P407, also known as PF-127, is a thermo-responsive synthetic polymer that has been widely explored in the preparation of in situ hydrogel. Due to its low mucoadhesiveness, poloxamer is usually used in combination with mucoadhesive polymers such as chitosan, carbopol and HPMC, to improve its mechanical and mucoadhesive properties. Among these, poloxamer and chitosan combination are mostly discussed in this review. Apart from its mucoadhesive properties, chitosan is capable of showing both thermo-responsive and pH-responsive characters [106,112,123]. Ionic cross-linking agents (e.g., β-glycerophosphate), and diluted acid (e.g., citric acid) are added to allow the gelation of chitosan, improve viscoelastic and mechanical performance of designed formulations [123,175,176,177]. These polymers are useful in the development of drug-delivery system due to their good biodegradability, biocompatibility, low toxicity, and good solubilizing capacity [112]. The drug-release behaviour of both stimuli-responsive formulations avoids accumulation and overdose of the antimicrobial drugs at the diseased site, diminishing the risk of drug resistance, tissue irritation and systemic adverse effects. Recently, thermo-responsive in situ hydrogels have been extensively studied for the development of favourable VDDS dosage form and have earned high commercial importance.

Apart from thermoresponsive polymers, pH-responsive characteristics of polymers are also encouraged in recent research. The proposed pH-responsive formulations mainly targeted high-risk women in the reproductive age, with a normal vaginal pH of 3.8 to 4.5. Nevertheless, it is reported that older adults aged 60 and above still actively engage in sexual activities. Lack of condom use due to their misconception of reduced fertility in later years and post menopause physiological changes (e.g., vaginal atrophy and dryness) increases the HIV-acquisition risk in older adults [178]. Therefore, formulations which are sensitive at higher pH (normal postmenopausal pH: around pH 6 to 7) should be developed to protect older generations from sexual transmission of HIV. Besides age, the physiological and pathological state of a person should be taken into account as these factors may attenuate the efficacy of the proposed formulations. Nonetheless, only sparse research is available for the pH-responsive systems in the treatment of vaginal infections. Moreover, pH responsiveness is particularly useful in vaginal microbicides for pre-exposure prophylaxis of HIV infections where a burst release of drugs depends on the exposure to semen. The high buffering capacity of semen increases vaginal pH to slightly alkaline, thus triggering pH-dependent mechanism, which can also be applied for the release of entrapped drug and to act to prevent transmission of HIV.

Alternatively, ion-responsive formulations are also studied for vaginal applications. Gellan gum is one of the promising in situ gelling polymers applicable in drug-delivery systems. They are also employed to enhance the mucoadhesion of Pluronic-based gels [179]. Mechanism of gelation of gellan gum involves the hydrogen bonding between cations and water via the formation of double-helical junction zones followed by aggregation of the double-helical segments. Cations play an important role in this mechanism. As compared to monovalent cations, gelation seems to be more effective in the presence of divalent cations [180]. The influence of ionic strength to gellan gum liquid-gel transition was discovered by Salunke et al. during their study on the mucoadhesive in situ gels for nasal administration [181]. The gelation period mainly depends on the concentration of the polymer used. Optimal concentration of the polymer is critical in the development of the in situ gels as it affects the quality of the gels. This is supported by Gupta et al. and Harish et al. in their studies on in situ gels [127,131,180]. A combination of gellan gum and other polymers such as HPMC and chitosan provides the formulation of a better mucoadhesive property and prolongs the drug release as shown in most of the studies.

In earlier sections, various combinations of stimuli, viz. temperature/pH, temperature/ion, temperature/enzyme, and pH/osmotic pressure were discussed to obtain superiority in the delivery systems. Among these, temperature/pH is the most widely explored stimuli combination. Temperature responsiveness is usually achieved using thermo-gelling polymers such as Pluronic and its derivatives as the gelation temperatures of these polymers are near to body temperature [182]. Similarly, the development of enzyme-responsive formulations for vaginal drug delivery is highly restricted for HIV infection prophylaxis. This review showed that some authors utilise two stimuli-responsive polymers, each responding to a different stimulus, in developing multi-stimuli responsive systems. Moreover, another technique is to chemically incorporate different responsive units into a single polymer building block. This technique not only preserves the properties of each responsive unit but also offers the fabrication of multi-stimuli responsive formulations in a more straightforward manner, thus negating the need to synthesise new types of block copolymer. One of the examples using this technique is the formation of poly(NiPAAm-co-BM-co-AA) by joining NiPAAm, BM, and AA via chemical linkages, thus generating a copolymer that responds to temperature and pH simultaneously.

The liquid crystalline precursor systems are employed nowadays to overcome the problem attributed to the gel-forming ability upon contact with the vaginal mucus. The precursor system exhibits isotropic characteristic and behaves as a Newtonian liquid able to flow out easily from the syringe facilitating vaginal application. Limited in vivo studies were done on the precursor system of liquid crystalline; thus, the drug-release kinetics under varying vaginal environment is not well understood. Further investigation is required to ensure the suitability and therapeutic efficacy of liquid crystalline precursor systems.

## 6. Conclusions

The vagina is established as a promising site of drug delivery in treating vaginal infections locally with recent approaches in vaginal drug delivery responding to the peculiarities of its anatomy and physiology. However, diverse aspects influencing the performance of drug delivery including the vaginal physiology and the highly fluctuating vaginal environment represent a challenge in the advancement of intravaginal formulations. The development of nanostructured systems for vaginal delivery as one of the novel approaches offers a broad array of advantages to tackle the drawbacks associated with the conventional intravaginal formulations that enhance therapeutic efficacy and patient compliance. Additionally, studies on vaginal delivery of nanocarriers showed encouraging outcomes in treating various vaginal microbial infections. This is determined by the composition, physicochemical features, and surface alteration or functionalization of the nanostructured systems. Despite these favourable outcomes, further research on animal models and toxicology assessment is essential to support the existing results obtained with each developed nanostructured system. Furthermore, studies in human volunteers are crucial to verify the safety and efficacy of the use of novel drug nanocarriers and verify definite patient acceptance and adherence.

A mucoadhesive system is a unique drug-delivery system that has been practised in various routes of drug administration for systemic and local effects. It has been employed in vaginal drug delivery in the form of vaginal films, vaginal tablets, vaginal gels as well as vaginal microparticles. The mucoadhesive property is achieved by incorporating the mucoadhesive polymers such as chitosan, gellan gum, HPMC, carbopol, and xanthan gum into the formulations. Based on the previous studies, it was proven that these polymers are biocompatible, non-toxic and safe to use as a drug carrier in drug-delivery systems. They could also provide better drug absorption by adhering to the mucosa for a longer period. Many marketed vaginal products have taken advantage of mucoadhesive polymers in order to achieve better therapeutic effects. The mucoadhesive VDDSs have potential for wider therapeutic uses than we know. Hence, they should be studied and developed in an advance manner to discover the potential use of the mucoadhesive VDDS.

Stimuli-responsive systems have gained considerable interest in vaginal drug delivery in recent years. The fascinating properties of stimuli-responsive polymers permit an optimal coverage of a low viscosity liquid over the mucosal surface upon administration and the formation of in situ gel in response to environmental stimuli. These approaches attain site-specific delivery and prolong the formulation residence time in the vaginal lumen, which ensures the achievement of better patient compliance. Numerous papers published in the literature justified that both thermal-responsive and pH-responsive systems are beneficial for improving the efficacy of the local application of drugs via the vaginal route. However, the ion-responsive system is considered new in the stimuli-responsive drug-delivery system. Only a limited number of studies and research have been done on the use of ion-responsive polymers in vaginal drug delivery. Advanced in vivo studies and clinical studies are needed as current studies are only up to in vitro evaluations. Therefore, extensive studies of the clinical applications and safety issues of the ion-responsive polymers would be valuable to further explore the roles of ion-responsive polymers in drug-delivery systems comprehensively.

Multi-stimuli responsive systems, which undergo chemical or physical changes in response to two or more environmental stimuli, have garnered enormous attention in drug delivery. Although many strategies and initiatives have been undertaken as demonstrated by the increasing number of scientific publications, clinical translation remains a challenge. The complicated synthesis of formulations to respond to multiple triggers may not be feasible for large-scale manufacturing. Moreover, variations in the vaginal environment during different life stages of women suggest the need for further research as the adaptation of more personalized regimens provide a better treatment of vaginal disorders. More experimental studies are required to assess the effectiveness, biocompatibility, and tolerability of the stimuli-responsive formulations in treating different types of vaginal condition at both preclinical and clinical stages.

## Figures and Tables

**Table 1:** Type, symptoms, underlying causes, risk factors, diagnosis and treatment of vaginal infections.

Type of Vaginal Infections	Causes	Risk Factors	Symptoms	Diagnosis	Treatment
Bacterial vaginosis (BV)	Caused by overgrowth of anaerobic and microaerophilic bacteria such as *Gardnerella vaginalis, Atopobium vaginae, Bacteroides* spp., etc.	Douching, sexual intercourse, and poor personal hygiene	Vaginal discharge with fishy odour, itchiness, and irritation	Nugent criteria, Amsel criteria, or Hay–Ison criteria	Oral metronidazole, oral clindamycin, oral tinidazole, metronidazole gel, clindamycin cream and clindamycin ovules
Vulvovaginal candidiasis (VVC)	Caused primarily by *Candida albicans*	Patient’s sexual and hygienic habits, the use of hormones and antibiotics, pregnancy, and immunosuppression	Abnormal vaginal discharge, dysuria, dyspareunia, and vaginal soreness	Positive wet-mount method, microscopic examination of vaginal swab culture, vaginal yeast count	Oral and topical azole therapies such as fluconazole, clotrimazole, miconazole, tioconazole, butoconazole and terconazole
Trichomoniasis	*Trichomonas vaginalis*	Infertility, poor pregnancy outcomes and sexually transmitted infections (STIs) acquisition	Yellow-green vaginal discharge, lower abdominal pain, dysuria, and vulvar irritation	Microscopic examination of vaginal fluid smear	Oral metronidazole and tinidazole, along with this topical formulation for metronidazole
Human immunodeficiency virus (HIV) infection	Human immunodeficiency virus	Risk of infection are associated with intravenous drug users, sex workers, transgender people, and, gay	Fever, myalgias, and swollen lymph nodes. Additionally liver dysfunction, tuberculosis, and acquired immunodeficiency syndrome (AIDS)	Detection of antibody	Antiretroviral drugs such as tenofovir, nevirapine, ritonavir, enfuvirtide, maraviroc etc.
Human papillomavirus (HPV) infection	Human papillomavirus	lower socioeconomic status, oral contraceptive use, history of multiple sexual partners, high parity, immunosuppression	Genital warts	Pap smear, biopsy	Prophylaxis by vaccine, treatment of wart by Salicylic acid, Trichloroacetic acid etc.

**Table 2:** Mucoadhesive polymeric approaches for vaginal drug delivery.

Type of Formulation	Objective of Research	Polymer Used	Disease Type/Drug	Cell Line/Animal Model	Outcomes	Source
Conventional liposomes	To develop azithromycin liposomes and evaluate their ability for the treatment of cervicovaginal infections	Phosphatidylcholine Hydrogenated phosphatidylcholine	*Escherichia coli*-related vaginal infections/azithromycin	HeLa cells	Ex vivo permeation study: 24% of drug accumulated on vaginal surface; 57% of drug retained within the vaginal tissue Minimum inhibitory concentration required to inhibit the growth of 50% of organisms ($MIC_{50}$): 2.48 µg/mL $IC_{50}$: 2.70 µg/mL Biocompatibility study: Cell viability remained above 70% at highest tested concentration (39.25 µg/mL)	[71]
Deformable propylene glycol liposomes	To develop azithromycin liposomes and explore their potentials for treating cervicovaginal infections	Phosphatidylcholine Monoacyl phosphatidylcholine Propylene glycol	*Escherichia coli*-related vaginal infections/azithromycin	HeLa cells	Ex vivo permeation study: 17% of drug accumulated on vaginal surface; 63% of drug retained within the vaginal tissue $MIC_{50}$: 2.49 µg/mL $IC_{50}$: 11.24 µg/mL Biocompatibility study: Cell viability remained above 70% at highest tested concentration (39.25 µg/mL)	[71]
Mucus-penetrating liposomes	To deliver the drug for local therapy for HPV vaginal infections	Cholesterol Methoxy poly (ethylene glycol)-modified lipids Phosphatidylcholine	HPV vaginal infections	-	Mucin binding: 7.0 ± 14.6% (pH 4.6) 3.8 ± 2.6% (pH 7.4)	[72]
Chitosan-coated liposomes	To prepare and optimize vaginal formulation of Resveratrol for effective treatment of vaginal infection and inflammation	Phosphatidylcholine Chitosan	Vaginal inflammation and infection/Resveratrol	-	SOD activity: increased by 26% compared to the controls (0.2% DMSO and empty liposomes)	[73]
Chitosan-surface modified PLGA nanoparticles	To vectorize clotrimazole with polymeric nanoparticles to treat vaginitis	Poly(lactic-co-glycolic acid) (PLGA) (lactide: glycolide 50:50) Low molecular weight chitosan	*Candida albicans* vaginal infections/Clotrimazole	Porcine cell culture	Mean zeta potential: +20.50 ± 0.20 mV In vitro release study: Burst release in the first hour (13%), release up to 35% in first 30 h, maximal amount of drug released at 432 h (18 days) was 99.2% MIC: *C. albicans*: 5 mg/mL Cell viability study: >80% at concentrations ≤ 100μg/mL	[74]
Chitosan-based nanoparticles	To evaluate the therapeutic efficacy of the developed miconazole nitrate-chitosan-based nanoparticles against murine vulvovaginal candidiasis	Chitosan	Vulvovaginal candidiasis/Miconazole	Mice bone marrow cells	Miconazole nitrate concentration: 63.9 mg/mL Antifungal activity: <1 × $10^{-5}$ CFU/g after seven days treatment Biochemical parameters: Blood urea nitrogen (BUN): 52.4 mg/dL Creatinine (Cr): 0.3 mg/dL Glutamic oxalacetic transaminase (GOT): 125.2 IU/L Glutamic pyruvic transaminase (GPT): 34.5 IU/L) Fractional DNA content (%): 2.4%	[75]
Eudragit RL100 nanoparticles coated with HA	To overcome the drawbacks of the conventional formulations with the developed AmB-loaded Eudragit RL100 nanoparticles coated with HA	Eudragit RL100 HA	Vulvovaginal candidiasis/AmB	-	In vitro release study: 28% and 81% of AMP were released in 24 h and 96 h, respectively Inhibition halos: 12.50 ± 1.37 mm Vaginal fungal burden (log CFU/mL): 0.00 ± 0.00 after 24 h	[77]
Nanocapsules	To develop chitosan nanocapsules loaded with antifungal drug suitable for vaginal application	Chitosan Lecithin	Vaginal candidiasis/Tioconazole, Econazole	Human keratinocyte cell line	Release assays over two days: Tioconazole: 21.8 ± 1.7 µg/mL Econazole: 5.6 ± 0.1 µg/mL Cell viability: ≥ 80% at concentrations ≤ 12 µg/mL for both tioconazole and econazole	[78]
Mucoadhesive liposomal gel	To fabricate sertaconazole nitrate-loaded liposomes for the effective treatment of vaginal candidiasis	Soy phosphatidylcholine Cholesterol Dimethyldidodecylammonium bromide (DDAB) Pectin	Vaginal Candidiasis/Sertaconazole	Sprague-Dawley rats	Zeta potential: +9.56 ± 0.34 mV In vitro drug release: 68.15 ± 1.35% Mucoadhesion evaluation: Drug retention in vaginal tissue 1.75 times more than control Ex vivo permeation study: drug penetrated was 2.47 times less than control In vivo evaluation: CFUs reduced from 72 ± 1.3 and 70 ± 1.9 to 42.4 ± 1.1 and 10 ± 1.9 in treated groups respectively.	[79]
Vaginal lipogel	To disperse drug-loaded liposomes into mucoadhesive gel for vaginal drug delivery	Carbopol^®^ 974P NF, HPMC K100M	Vaginitis/Benzydamide hydrochloride	-	Drug diffusion study Carbopol^®^ 974P NF based: 57.4 ± 6.15% released in 24 h HPMC K100M based: 67 ± 4.5% released in 24 h Mucoadhesive studies: Work of mucoadhesion Carbopol^®^ 974P NF based: 0.176 ± 0.037 mJ/cm^2^ HPMC K100M based: 0.243 ± 0.053 mJ/cm^2^	[80]
Vaginal in situ gel	To combine poloxamer and different types of HPMC to improve the mucoadhesive and mechanical properties of the in situ gels to prolong the residence time in vaginal cavity.	HPMC K100M, E50	Vaginal candidiasis/ Clotrimazole	-	Gelation temperature: 34.47 ± 0.014–34.50 ± 0.026 °C Gelation time: 326.746 ± 0.123–326.883 ± 0.025 s Mucoadhesion study: 0.043 ± 0.011–0.082 ± 0.040 mJ Drug release study: HPMC K100M based: 74.755% after 6 h, 86.082% after 24 h. HPMC E50 based: 87.866% after 6 h, 100% after 24 h. Microbiological study: Inhibition zone of loaded HPMC K100M: 22 ± 0.5mm Inhibition zone of loaded HPMC E50: 20 ± 1.0 mm In vivo distribution study: The formulations remained on the vaginal mucosa for 24 h after application and no significant difference was found among the formulations.	[82]
Microgel	To formulate and evaluate mucoadhesive microgels for enhanced antifungal activity of miconazole nitrate.	Polycarbophil	Vulvovaginal candidiasis/Miconazole nitrate	Female rabbits	Ex vivo mucoadhesive strength: 12.02 ± 1.05 dyne/cm^2^ In vitro drug release: Microgel containing the least amount of PEG 4000 showed the best controlled release property. In vivo anticandidal properties: reduce the level of infection to 13.94% at the end of the treatment Vaginal tolerance and histopathological studies: The microgel did not affect the morphology of vaginal tissue after 14-day dosing.	[81]
Nanocapsules in hydrogel	To design polymeric nanocapsules containing hydrogel to increase the drug residence time in the vaginal epithelium.	Eudragit^®^ RS100 nanocapsules, gellan gum	Vaginal trichomoniasis/Indole-3-carbinol (I3C)	-	Nanocapsules suspension caused a greater reduction in the trophozoites viability (*p* < 0.001). I3C nanocapsules IC50 = 2.09 µg/mL I3C hydrogel presented higher spreadability factor (*p* < 0.05) in relation to hydrogels containing nanocapsules. Rheological evaluation: The hydrogel showed non-Newtonian flow. Irritation studies: No sign of irritation was observed for nanocapsule suspensions and semisolid vehicle. Mucoadhesion studies: Nanocapsule hydrogel formulations presented higher mucoadhesive force than the others (*p* < 0.001).	[83]
Nanoemulsion gel	To formulate mucoadhesive nanoemulsion-based vaginal gel for vaginal candidiasis.	Carbopol 934, HPMC, NaCMC, xanthan gum	Vaginal candidiasis/Oxiconazole nitrate	-	In vitro bioadhesion study: The nanoemulsion gel showed significantly higher residence time as compared to Tinox^®^ cream (*p* < 0.05). In vitro antifungal activity: Drug-loaded nanoemulsion gel exhibited stronger antifungal activity than the Tinox^®^ cream (*p* < 0.05). In vitro release study: Xanthan gum containing formulations showed controlled release properties.	[84]
Electrospun nanofibers	To develop and characterize innovative vaginal dosage forms for the treatment of bacterial vaginosis	PVP	Bacterial vaginosis/Metronidazole	-	Work of mucoadhesion: 2250–2690 $\mathrm{mJ}\cdot m^{-2}$ In vitro release study: >80% of MTZ was released in five minutes Permeability coefficient: 10% PVP: $1.0\times 10^{-2}\mathrm{cm}\cdot h^{-1}\pm 2\times 10^{-3}$ 12.5% PVP: $19\times 10^{-3}\mathrm{cm}\cdot h^{-1}\pm 5\times 10^{-3}$ 15% PVP: $11\times 10^{-3}\mathrm{cm}\cdot h^{-1}\pm 1\times 10^{-3}$	[86]
Electrospun mucoadhesive nanofibers	To assess the antifungal properties of mucoadhesive clotrimazole loaded nanofiber versus vaginal film	Dextran Sodium alginate Polyvinyl alcohol	Vaginal candidiasis/Clotrimazole	Human gingival fibroblast cells	Swelling capacity: 88.84% Mucoadhesive: 1.35g MIC range: 125–250 μg/mL MTT assay: cell viability remained above 70% at concentration of 5, 10 and 20 μg/mL at 24 h	[87]
PLGA nanofibers	To develop AmB-loaded PLGA nanofibers as alternative drug delivery systems	PLGA (75:25)	Vulvovaginal candidiasis/AmB	Female Wistar rats (*Rattus novergicus*)	In vitro release study: 12.5% of AmB was released in 24 h; cumulative drug release achieved 100% on the eighth day; no initial or final burst release MIC range: 0.5–1.0 μg/mL In vivo fungicidal activity: 40% of the rats remained infected after six hours of local treatment and complete eradication of infection in all rats after 72 h of local treatment.	[88]
Vaginal film	To develop a mucoadhesive vaginal film based on a mixture of chitosan (CHI) and poly(2-ethyl-2-oxazoline) (POZ).	Chitosan	Bacterial vaginosis/ Ciprofloxacin	-	Surface pH: 3.76–3.86 In vitro drug release: 56 ± 1% CHI/POZ (40:60) Growth inhibition zones: *E. coli* ATCC 8739: 39.3 ± 2.3 mm to 42.5 ± 2.0 mm *S. aureus* ATCC 6538-p: 36.0 ± 1.9 mm to 46.1 ± 1.7 mm chitosan based film exhibit strongest mucoadhesive property.	[93]
Vaginal membrane	To develop an alginate/chitosan (AC) membrane with bactericide effect and controlled release property.	Alginate, chitosan	Bacterial vaginosis/Metronidazole	Cervix epithelial cells Ect1/E6E7	Work of mucoadhesion of AC membrane: 0.497 ± 0.215 mJ Membrane dissolution study: 50% of weight remained after 30 days Biocompatibility assay: No significant reduction of viability with respect to the control cells both after 24 h and 48 h. Antimicrobial activity: Both AC and chitosan membranes reduced the CFU/mL count by >4 Logs and >3 Logs for *S. aureus* and *G. vaginalis,* respectively.	[94]
Vaginal film	To combine the advantages of in situ gelling polymers and thiomers to enhance vaginal residence time.	S-protected gellan gum (S-GG 81, S-GG 174)	Bacterial vaginosis/Metronidazole	Caco-2 cells	Biocompatibility: Cells remained viable to >87% after incubation of 3 h and 24 h. (S-GG concentration: 0.5% *m*/*v*) Swelling study: S-GG 81: gained 6.4 times of its initial weight S-GG 174: gained 11 times of its initial weight Viscosity: S-GG 81: increased up to 1.84-fold S-GG 174: increased up to 4.3-fold Ex vivo mucoadhesive study: S-GG 81: improved by 1.5-fold S-GG 174: improved by 3-fold, >60% of S-GG 174 remained on the vaginal mucosa after 3 h Drug release study: S-GG 81: 89% within 3 h S-GG 174: 77% within 3 h Growth inhibition zones: Metronidazole (control): 30 ± 1 mm S-GG 81: 17 ± 0.3 mm S-GG 174: 16 ± 1 mm	[95]
Vaginal film	To develop a vaginal film using of two polymers to enhance the therapeutic efficacy for vaginal candidiasis.	Chitosan, HPMC	Vaginal candidiasis/Ticonazole	Human HCC cell lines Huh7	Swelling study: CH-based films swelled more than the CH/HPMC based films. Mucoadhesive study: No significant differences among the formulated films (*p* > 0.01). Dissolution study: The films showed fast drug release, reaching almost 100% after 60 min. Biological activities: Time-kill: Unloaded films: When chitosan was used at concentrations higher than 1.0 mg/mL, it possessed fungistatic and fungicidal activities. Ticonazole loaded films: The fastest activity was observed in chitosan based film (15 min) and chitosan/HPMC-based films (30 min). Cytotoxicity study: Films based on 100% chitosan induced a 35–54% reduction in cell viability after 24 h of incubation. No toxicity effect was observed in both loaded and unloaded films based on chitosan/HPMC.	[96]
Vaginal film	To develop vaginal films with a different combination of polymers and plasticizers for vaginal candidiasis.	HPC, sodium alginate	Vaginal candidiasis/Clotrimazole	-	Ex vivo mucoadhesion strength: 0.705 N In vitro antifungal activity: 24 mm on *C. albicans* (higher than marketed Candid-V6^®^ tablet) 8 mm on *Lactobacillus* sp. (did not inhibit the growth of *Lactobacillus* sp.) Percentage of swelling: 60% (nearly similar to marketed Candid-V6^®^ tablet) In vitro dissolution studies: 70% drug release in 1 h and 83% drug release in 6 h	[97]

**Table 3:** Thermo-responsive systems for vaginal drug delivery.

Type of Formulation	Objective of Research	Stimuli Type and Polymer Used	Disease Type/Drug/Formulation	Cell Line/Animal Model	Outcomes	Source
Thermal gelling foam aerosol	To combine the advantages of gel and foam for retention and drug penetration in vagina	Temperature Polymers: P407 P188 Carbopol	Vaginitis and cervical erosion/silver nanoparticle	Sprague Dawley (SD) rats	Gelation temperature: 35.7 ± 0.3 °C Gelation time: 8.7 min Expansion foam height: 64.8 ± 2.5 mm Foam duration time: 123.2 ± 5.6 min Extended drug release over four hours, with rapid release within the first hour MIC: *E. coli* and *S. aureus*: 25 μg/mL, *P. aeruginosa*: 12.5 μg/mL, *C. albicans*: 100 μg/mL (most resistant) Vaginal epithelial hyperkeratosis, light uterine eosinophilic infiltration, and local infiltration of lymphocytes Comprehensive irritation index: 2.66	[111]
In situ poloxamer-chitosan hydrogel	To develop a vaginal delivery system for benzydamine hydrochloride (BNZ) using poloxamer-chitosan in situ hydrogels.	Temperature Polymers: P407 Chitosan H	Vaginitis/benzydamine hydrochloride	Cow vaginal mucosa	Gelation temperature: >31.8 ± 0.6 °C pH: 3.9 Viscosity: 949 ± 5 cP Mucoadhesion values: F_max_: 0.188 ± 0.021 N, W_ad_: 0.997 ± 0.059 Nmm, W_mucoad_: 0.316 ± 0.031 mj/cm^2^ Drug release at 6h: 65.3 ± 5.1% Mechanical properties: Hardness: 0.310 ± 0.075 N, adhesiveness: 0.431 ± 0.052 N.mm, cohesiveness: 0.711 ± 0.047, compressibility: 1.174 ± 0.218 N.mm and elasticity: 0.739 ± 0.028	[112]
In situ gel of tinidazole	To avoid hepatic first-pass metabolism, a reduction in the incidence and severity of gastrointestinal side-effects.	Temperature Polymers: P407 HPMC E100	Bacterial vaginosis/Tinidazole	-	Spreadbility: 15mm Mechanical properties: Hardness: 0.012 ± 0.000 N, compressibility: 0.025 ± 0.001 N.mm, adhesiveness: 0.038 ± 0.002 N.mm, elasticity: 0.800 ± 0.036, cohesiveness: 0.574 ± 0.069 Cumulative drug release: After 1h: 30.5%, after 4h: 53.6%, after 8h: 71.3% Zone of inhibition: *E. coli*: 31 mm, *S. aureus*: 31 mm Mean Hen’s Egg-Chorioallantoic Membrane (HET-CAM) test score: Up to 240 min: 0, 480 min: 0.33, 1400 min: 0.66	[115]
Clotrimazole-loaded vaginal gel	To formulate and evaluate thermoreversible gel of antifungal agent Clotrimazole for treatment of vaginal infection.	Temperature Polymers: Pluronic F127 Pluronic F68 Polycarbophil	Candidiasis/ Clotrimazole	-	Gelation temperature: 31 °C to 32 °C Spreadability: 2.56 s to C: 2.53 s Gel strength: 51.3 s to 55.5 s Mucoadhesive strength: 2.02 to 2.38 dyne/cm^2^ Drug diffusion after 11 h: 95.2% to 98.5%	[116]
Thermoreversible gel-loaded AmB	To achieve an improved release of amphotericin B for skin and vaginal treatment against *Candida* spp.	Temperature Polymer: P407	Candidiasis/AmB	Vaginal porcine mucosae	Viscosity: 4 °C: 90.62 ± 7.112 × 10^−2^ mPa·s, 32 °C: ~9.8 mPa·s Ex vivo permeation studies: Receptor chamber: No AmB detected In the vaginal mucosa: Retained 737.52 μg/g/cm^2^ MIC: *C. albicans*: 0.09 μg/mL, *C. glabrata*: 0.37 μg/mL, *C. parapsilosis*: 0.19 μg/mL	[117]
Miconazole nitrate liquid vaginal suppository	To prepare and evaluate of in situ gelling miconazole nitrate liquid vaginal suppository for fungal infection.	Temperature Polymers: P188 P407 HPMC	Candidiasis/Miconazole nitrate	-	Gelation temperature: 34 °C pH: 7.42 Gel strength: 25.2 ± 0.3 s Mucoadhesive force: 77.2 ± 0.2 dyne/cm^2^*10^2^ In vitro drug release in 8h: 87.05 ± 0.04% Inhibition zone: 50.22 ± 0.12 mm	[118]
Auranofin-nanoparticle composite hydrogel	To address the challenges of vaginal drug delivery with a novel thermo-responsive nanoparticle (NP)-hydrogel composite for the effective topical treatment of trichomoniasis.	Temperature Polymers: Chitosan Pluronic F127 (P407)	Trichomoniasis/Auranofin	Bagg Albino (BALB/c) mice	EC_50_: 22 μM Vaginal AF level: 0.5–1 μg per g tissue	[119]
Tenofovir-loaded vaginal gel	To design a thermogelling system which releases the antiretroviral agent in a controlled fashion while patient compliance will be increased with both prolonged contact time and ease of application.	Temperature Polymers: Pluronic F127 Chitosan	HIV infection Drug: Tenofovir	L929 cell line	Gelation temperature: 26.2 ± 0.2 °C Viscosity: At 25 °C: 163.3 Poise, At 35 °C: 1002.4 Poise Mucoadhesion: 0.516 ± 0.136 N·s at 37 °C Burst release effect: First 0.5 h: Released approximately 27% of TFV, by the end of 24 h: Released 85% of TFV Cell viability: High Chitosan: Non-toxic at concentration up to 1 mg/mL Pluronic F127: 75–95% cell viability at concentration range of 2.5–0.625%	[120]
Polymeric blend hydrogel	To fabricate and optimize different thermo-responsive and mucoadhesive hydrogel for improving the vaginal delivery of CUR.	Temperature Polymers: P407 Chitosan HPMC K4M	Vaginal mucosal inflammation and infectious diseases, including HPV infection/Curcumin	HeLa cells	pH: 4.54 ± 0.03 Gelation temperature: 32.8 ± 0.55 °C Erosion rate: 51.3% of the initial hydrogel weight after 90 min Mucoadhesion properties: Maximum force for separation: 8.53 ± 0.41 g, work of adhesion: 243.61 ± 10.65 g s^−1^, deformation of peak: 2.17 ± 0.14 mm Drug release: 52% of CUR released over 180 min Percentage of antioxidant activity at the end of 60 min: 76.5%	[121]

**Table 4:** pH-responsive systems for vaginal drug delivery.

Type of Formulation	Objective of Research	Stimuli Type and Polymer Used	Disease Type/Drug	Cell Line/Animal Model	Outcomes	Source
Freeze-dried bigels	To obtain freeze-dried bigels for the controlled release of Tenofovir (TFV) in the vaginal environment.	pH Polymers: Pectin	HIV infection/Tenofovir	-	Hardness: 6.48 ± 0.26 N Mucoadhesion work: 0.20 ± 0.02 N Swelling profile: Vaginal pH: Experienced swelling until 24 h followed by loss of structure Drug release: Acidic pH: Released about 60% of the dose in the first 6h Alkaline pH: Released about 90% of the dose in the first 6h	[122]
Vaginal polyelectrolyte layer-by-layer film	To develop polyelectrolyte multilayers vaginal films based on optimized chitosan derivatives and Eudragit^®^ S100 using the layer-by-layer technique.	pH Polymers: Chitosan citrate Methyl polymethacrylate (Eudragit^®^ S100)	HIV infection/Tenofovir	Lymphoblastic cell line (MT-2) Macrophage-monocyte derived cell line (THP-1) Uterine/endometrial epithelial cell line (HEC-1A)	pH: 2–3 Mucosal adhesiveness: Remained mucoadhered for 120 h Drug release: Acidic pH: Release up to 120 h Alkaline pH: Release up to 4 h Cytotoxic concentration 50 (CC_50_): Around 1000 μg/mL	[123]
Polyurethane hydrogel in intravaginal ring	To synthesise supramolecular pH-responsive hydrogel to filled in the lumen of reservoir-IVRs for the on-demand release of nanocarriers.	pH Polymers: 2,2-dimethylolpropionic acid (DMPA) Hexamethylene diisocyanate (HDI) PEG	HIV infection/siRNA	VK2/E6E7 cell lines	Zeta potential of the nanoparticles: pH 3.5: −6 ± 1 mV, pH 4.5: -2.1 ± 0.1 mV, pH 7.0: −1 ± 2 mV In vitro release: pH 4.2: close-to-zero release for 12 h, 13.7 ± 0.5% for 12–24 h, pH 7.0: 42 ± 7% for 24 h Nanoparticles showed no toxicity, whereas PU hydrogel showed low toxicity	[124]
Polyurethane membrane in intravaginal ring	To develop a pH-responsive membrane for vaginal delivery of nanoparticles to achieve site specific delivery.	pH Polymers: 1,4-Bis(2-hydroxyethyl) piperazine (HEP) 4,40-Methylenebis (phenyl isocyanate) (MDI) PEG	HIV infection/siRNA	Human vaginal epithelial cell line (VK2/E6E7) Human T-cell line (Sup-T1)	Pore size: pH 4.5: 1.8 ± 0.6 μm, pH 7.0: 2.2 ± 0.6 μm Diameter of fibre: pH 4.5: 1.4 ± 0.5 μm, pH 7.0: 1.2 ± 0.5 μm Thickness: pH 4.5: 50 ± 2 μm, pH 7.0: 35 ± 1 μm Zeta potential: pH 4.5: 14.2 mV, pH 7.0: 2.2 mV Viscosity: 0.306 ± 0.003 Pa·s In vitro release: pH 4.5: close-to-zero release, pH 7.0: sustained release (increased to 60 ± 6% after 24 h) Non-toxic and did not induce an inflammatory microenvironment	[125]
Synthetic mucin-like polymer system	To design a biologically inspired synthetic mucin-like polymer system to prevent transport of virions in the vagina.	pH Polymers: Phenylboronic acid (PBA)-salicylhyldroxamic acid (SHA) crosslinked polymers	HIV infection	VK2/E6E7 cell lines/ VEC-100 tissues BALB/c mice	Relaxation/lifetime: pH 4.8: 0.9s, pH 7.6: >60 s Below critical shear rate (<0.6 s^−1^): Shear thinning behaviour Above critical shear rate (<0.6 s^−1^): Shear thickening behaviour Migration: HIV virions: decreased with the increased in pH, Macrophages: independent of dilution and remained confined within the first 500 μ in the gel In vitro safety evaluation: Cell viability: ~80–90%, tissue viability: 98 ± 12%, in vivo safety assessment: 81 ± 0.8% Ex vivo safety assessment: vaginal tissues appeared normal with intact lamina propia and sub-mucosa. Some locations showed signs of vacuolated and necrotic cells	[126]

**Table 5:** Ion-responsive systems for vaginal drug delivery.

Type of Formulation	Objective of Research	Stimuli Type and Polymer Used	Disease Type/Drug	Cell Line/Animal Model	Outcomes	Source
Vaginal in situ gel	To develop an in situ gel of clindamycin with prolonged retention time in the vaginal cavity.	Ion-responsive Polymer: Gellan gum	Bacterial vaginosis/Clindamycin	-	A concentration of 1% of both chitosan and gellan gum formed colorless and transparent gel with good gelling capacity. Physicochemical properties: Clear solution, pH: 5.0–5.5, mucoadhesive force: 0.118 N, and retention time: 98 min The formulations showed a pseudoplastic flow which is desirable. The developed formulation displayed 32.3% cumulative drug release after 2 h, 77.4% after 6 h and 97.2% after 12 h. Chitosan/gellan gum based formulation was non-irritant to mild irritant (well tolerated). After 6 months of the test, overall degradation is <5%.	[127]
Vaginal in situ gel	To combine advantages of both gels and solution for vaginal application.	Ion-responsive Polymer: Gellan gum (Gelrite^®^)	Vaginitis/Clindamycin	-	Physicochemical properties: pH values: 5.3–5.5, gelling temperature: 25.4–36.8 ºC, viscosity: 40 ± 0.12–90 ± 0.18 cps, spreadability: 14–25 mm The developed formulation displayed 33.3% cumulative drug release after 2 h, 67.4% after 6 h and 98.9% after 12 h. The drug loaded formulation (1% *w/v* HPMC and gellan gum respectively) showed zone of inhibition of 18 mm. HPMC/gellan gum based formulation was non-irritant to mild irritant (well tolerated). The developed formulation remained stable in physicochemical properties after 3 months of the test.	[130]
Vaginal in situ gel	To formulate an in situ vaginal gel based on ion-activated systems to prolong the drug release.	Ion-responsive Polymer: Gellan gum	Vaginal trichomoniasis/Secnidazole	-	Physicochemical properties: pH value: 4.1–4.5, viscosity: 11–16 Pa·s, spreadability: 25.2–30.7 g·cm/s, mucoadhesive force: 52.2 ± 2.40–56.3 ± 5.67 dyn/cm^2^, gel strength: 0.5–7.2 g Optimized formulation: Drug release showed 80% release profile up to 360 min. Release kinetic indicating Fickian diffusion release mechanism.	[131]

**Table 6:** Multi-stimuli responsive systems for vaginal drug delivery.

Type of Formulation	Objective of Research	Stimuli Type and Polymer Used	Disease Type/Drug	Cell Line/Animal Model	Outcomes	Source
In situ gel	To develop in situ gel formulation for prolonged drug release for vaginal application.	Temperature and ion Polymer: PF-127/68 and gellan gum	Bacterial vaginosis/Clindamycin	-	The in situ gel showed prolonged muco-adhesion. 97.5% drug released after 9 h, showing prolonged release.	[133]
Hydrogel	To develop biocompatible hydrogel-based formulations for sustained drug release.	Temperature and pH Polymer: SA/pNIPAAm	Bacterial vaginosis/Oxytetracycline (OTC)	Human umbilical vein endothelial cells (HUVEC) cells	Initial slow release of OTC in the pH range from 1.2 to 11.65 during the initial 24 h, and then the release was maintained in a range of 40–45 μg·mL^−1^ during 72 h. After 72 h, the increased OTC release may be responsible the augmented SA/pNIPAAm hydrogel degradation. The OTC-loaded hydrogel (50.11 µg mL^−1^) showed high cell viability of 175 % on HUVEC cells.	[140]
Flakes-loaded in situ gel	To design and develop a multifunctional ketoconazole (KTZ) carrier to provide efficient spreading and coating of the vagina due to free-flowing properties during application, flakes entanglement within folded vaginal epithelia, and sustained release for the treatment of VVC.	Temperature and ion Polymer: PF-127 and chitosan/gellan gum	Vulvovaginal candidiasis/KTZ (in the form of KTZ/β-CD complex)	-	Initial burst release (39 ± 3%) during the first hour, followed by sustained release to reach 91.7 ± 1.6% after 6 h. Larger inhibition zones against *C. albicans* compared to KTZ suspension and marketed terconazole vaginal cream “0.8% Gynoconazol^®^”.	[143]
In situ gel	To design a vaginal gel formulation with thermosensitive and mucoadhesive properties to ensure longer residence at the infection site, thereby providing a pH-dependent sustained release profile for AmB.	Temperature and pH Polymer: MBCP-2 copolymer	Vulvovaginal candidiasis/AmB (in the form of AmB-HPγCD complex)	HEK 293 cells and female ICR mice	Constant release of AmB for 3 days at pH 5.0 All concentrations of AmB-HPγCD complex-loaded gel showed no cytotoxicity. No visible sign of inflammation or necrosis in vaginal mucosa of female ICR mice after repetitive vaginal application of 20 μL 25% MBCP-2 copolymer solution 3 times per week over 2 consecutive weeks.	[146]
In situ hydrogel	To prepare and characterise physically crosslinked gel formulations of chitosan-graft-PNIPAAm and PVA for smart delivery of voriconazole for mucosal applications.	Temperature and pH Polymer: CS-*g*-PNIPAAm/PVA grafted copolymer (75/25 ratio)	Drug: Voriconazole	HK-2 and NIH-3T3 cell lines	Flow behaviour: non-Newtonian Viscoelastic behaviour: Pseudoplastic Initial burst release (23.6%) during the first 30 min, followed by sustained release to reach 38% after 8 h. Showed no cytotoxicity on both cell lines.	[138]
In situ hydrogel	To design a smart bio-responsive microbicide that is able to coat vaginal tissue and provide a burst release of entrapped antiviral agents when exposed to semen.	Temperature and pH Polymer: Poly(NiPAAm-co-BM-co-AA)	HIV infection (Pre-exposure prophylaxis)	Immortalised L-929 mouse fibroblasts cell line	Negligible gel erosion in the presence of VFS (pH 4.2). Rapid erosion of gel in the presence of SFS (pH 7.7). In VFS (pH 4.2), drug release profile showed 25 ± 5% within the first 100 min. In VFS diluted with SFS (1:4 dilution), there was a significant increase to 49 ± 3% within 5 min and to a maximum of 81 ± 1% within an hour. The hydrogel showed a semen-triggered burst release of drug. Poly(NiPAAm-co-BM-co-AA) terpolymer (IC_50_ of 30 mg/mL) was less cytotoxic compared to Carbopol 974 (IC_50_ of 5 mg/mL). Poly(NiPAAm-co-BM-co-AA) terpolymer at 70 mg/mL has equal biocompatibility as Carbopol 974 at 0.5–20 mg/mL.	[150]
Organogel	To develop and evaluate HA/palm oil-based organogel loaded with MRV which would be released using hyaluronidase as the trigger for pre-exposure prophylaxis of HIV.	Temperature and hyaluronidase enzyme Polymer: HA	HIV infection (Pre-exposure prophylaxis)/Maraviroc	TZM-bl cell lines and HeLa cell lines	2.5-fold increase in the release of MRV in the presence of hyaluronidase enzyme compared to no enzyme. Thus, MRV would be released from the optimised organogel upon trigger by hyaluronidase. The optimised organogel inhibit HIV-1 infectivity on TZM-bl cell lines. The optimised organogel preserved the viability of *L. crispatus* and showed no cytotoxicity in HeLa cells for 14 days in vitro.	[151]
pH-sensitive vaginal osmotic pump	To develop an osmotic pump tablet that can deliver antiretrovirals for several days.	pH and osmotic pressure Polymer: CAP	HIV infection (Pre-exposure prophylaxis)/ IQP-0528	Adult female sheep	97% of drug released after 12.5 days, thus showing sustained release. 4.6 ± 1% drug was released after a day, reaching about 47.1 ± 3.3% at day 10, thus showing multi-day sustained release.	[157]
In situ liposome gel	To prepare a dual temperature- and pH-sensitive cleavable liposome gel loaded with arctigenin.	Temperature and pH Polymer: P407/188 and mPEG-Hz-CHEMS	Arctigenin	HEK 293 cells	Constant release of arctigenin for 3 days at pH 5.0. Arctigenin-loaded dual-sensitive liposome gel showed less cytotoxic effect than free arctigenin. The most diluted sample of arctigenin-loaded dual-sensitive liposome gel showed lowest cytotoxic effect among all formulations containing arctigenin.	[159]

**Table 7:** Liquid crystalline precursor systems for vaginal drug delivery.

Type of Formulation	Objective of Research	Stimuli Type and Polymer Used	Disease Type/Drug	Cell Line/Animal Model	Outcomes	Source
Liquid crystal precursor mucoadhesive systems	To evaluate the potential of the methanolic extract of scapes of *Syngonanthus nitens* (*S. nitens*) and a *S. nitens*-loaded liquid crystal precursor system	Water Carbopol 974P Polycarbophil	Vulvovaginal candidiasis with *Candida krusei* Drug: *Syngonanthus nitens*	Wistar female rats (*Rattus* *norvegicus*)	Mucoadhesive force: F: 5 mN, F100: 12 mN MIC range: 31.2–62.5 µg/mL Inhibition biofilm assay: 10 mg/mL	[162]
Liquid crystal precursor mucoadhesive systems	To evaluate the antifungal activity of *S. nitens* extract that was not loaded or loaded into a liquid crystal precursor system	Water Carbopol 974P Polycarbophil	Vulvovaginal candidiasis with *Candida albicans* Drug: *Syngonanthus nitens*	Wistar female rats (*Rattus* *norvegicus*)	Mucoadhesive force: S: 6mN, S100: 13 mN MIC range: 31.2–62.5 µg/mL Inhibition biofilm assay: 1.25-20.0 mg/mL	[163]
Phytantriol-Based in situ liquid crystal gel	To evaluate the potential of in situ liquid crystal gels based on phytantriol for vaginal delivery	Water Phytantriol 64% *w*/*w* Ethanol 16% *w*/*w* Water 20% *w*/*w*	Cervical cancer Drug: Sinomenine hydrochloride	Sprague Dawley (SD) rats	Minimum volume of vaginal fluid simulant (VFS): 64.56 ± 3.26 μL Minimum time for phase conversion: 3.92 ± 0.38 s Irritation score: 1.10 ± 0.23	[164]
Mucoadhesive in situ gelling liquid crystalline precursor system	To improve the vaginal administration of drugs	Water P407	Vaginal bacterial infection and gynaecological cancers. Drug: Hypericin	L-929 cell line	Mucoadhesive strength: 26 mN upon dilution Cell viability: above 80% upon dilution	[165]

## Data Availability

The data presented in this study are openly available.
